# Leveraging 3D Model Systems to Understand Viral Interactions with the Respiratory Mucosa

**DOI:** 10.3390/v12121425

**Published:** 2020-12-11

**Authors:** Ethan Iverson, Logan Kaler, Eva L. Agostino, Daniel Song, Gregg A. Duncan, Margaret A. Scull

**Affiliations:** 1 Department of Cell Biology and Molecular Genetics, Maryland Pathogen Research Institute, University of Maryland, College Park, MD 20742, USA; iverson@umd.edu (E.I.); eagostin@umd.edu (E.L.A.); 2 Biophysics Program, University of Maryland, College Park, MD 20742, USA; likaler@umd.edu (L.K.); gaduncan@umd.edu (G.A.D.); 3 Fischell Department of Bioengineering, University of Maryland, College Park, MD 20742, USA; bsong12@umd.edu

**Keywords:** 3D model, tissue engineering, mucus, periciliary layer, mucosal barrier, viral infection, microscopy

## Abstract

Respiratory viruses remain a significant cause of morbidity and mortality in the human population, underscoring the importance of ongoing basic research into virus–host interactions. However, many critical aspects of infection are difficult, if not impossible, to probe using standard cell lines, 2D culture formats, or even animal models. In vitro systems such as airway epithelial cultures at air–liquid interface, organoids, or ‘on-chip’ technologies allow interrogation in human cells and recapitulate emergent properties of the airway epithelium—the primary target for respiratory virus infection. While some of these models have been used for over thirty years, ongoing advancements in both culture techniques and analytical tools continue to provide new opportunities to investigate airway epithelial biology and viral infection phenotypes in both normal and diseased host backgrounds. Here we review these models and their application to studying respiratory viruses. Furthermore, given the ability of these systems to recapitulate the extracellular microenvironment, we evaluate their potential to serve as a platform for studies specifically addressing viral interactions at the mucosal surface and detail techniques that can be employed to expand our understanding.

## 1. Introduction

Through the simple act of breathing, the respiratory tract is exposed to the external environment, and therefore represents both an important target and portal for infection. Respiratory viruses comprise many clinically significant pathogens, including influenza virus, respiratory syncytial virus, and severe acute respiratory syndrome coronavirus 2 (SARS-CoV-2), among others, for which our knowledge of infection mechanisms is incomplete. However, the lung is a complex and dynamic tissue characterized by different anatomical zones comprising different cell types, a temperature gradient, and the continuous forces exerted by mechanical clearance mechanisms and tidal breathing. As a mucosal tissue, the airway epithelium also evolved a physical and chemical extracellular barrier that promotes normal tissue homeostasis and neutralizes pathogens. While immortalized cell lines have historically been the workhorse of viral research, they lack critical aspects of the airway microenvironment at both the cellular and extracellular level. In addition, animal and human challenge studies are limited by species-specific differences or the types of analyses that can be performed. Thus, understanding respiratory virus–host interactions in the natural infection setting is a challenge. While no model system is perfect, Transwell cultures of airway epithelium at air–liquid interface (ALI), organoid models, and tissue-engineered platforms offer tractable systems to dissect the mechanisms of infection in the native context. By virtue of their improved recapitulation of the extracellular mucosal compartment, these culture models exhibit characteristics—termed “emergent properties”—not seen in more reductive systems. These models were initially pioneered in the 1980s [1,2,3,4,5] and have since been further developed and utilized by many laboratories to uncover novel viral phenotypes. Here we provide an overview of these complex in vitro airway models and highlight their successful application in virology.

Furthermore, given the ability of these models to recapitulate the mucosal surface, in parallel with the development of genetic tools, virus-labeling techniques, and advanced microscopy applications, we ask whether these systems represent useful platforms to probe viral interactions in the extracellular space. Viral penetration of the secreted mucus barrier is essential for infection and the importance of mucosal barriers and host factors within mucus in viral infection has been recognized for decades [6,7,8]. Still, how viruses overcome this barrier, what specific host components in this extracellular microenvironment impact infection, and conversely, how infection alters the mucosal barrier remain critical open questions in understanding respiratory viral pathogenesis. Moreover, an accurate understanding of altered barrier state observed in chronic lung disease and how this influences viral infection phenotypes is essential for understanding why some individuals may be at greater risk of infection. Thus, in the latter half of this review, we summarize host factors comprising the extracellular barrier, evaluate the utility and potential application of 3D models in answering the questions above, and highlight tools and techniques that can be utilized to do so.

## 2. In Vitro Airway Models with Emergent Properties

### 2.1. Air–Liquid Interface Transwell Systems

**Figure 1:**
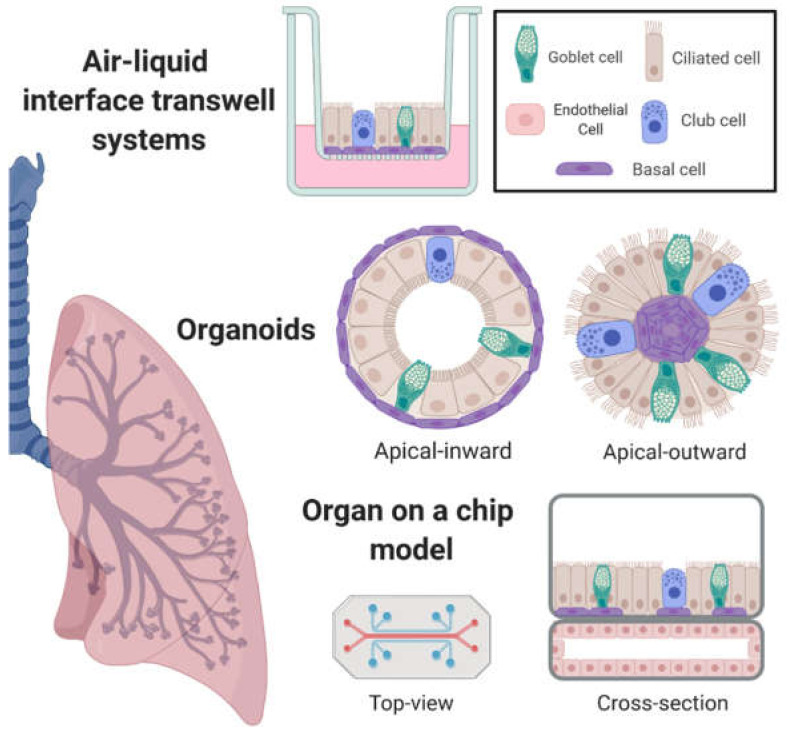
In vitro model systems of the human respiratory tract with emergent properties. Air liquid interface (ALI) systems utilize porous membranes for cell attachment, giving rise to consistent open-air lumen development. Organoid systems self-organize within a matrix or scaffold, often leading to variable orientations of the lumen. Organ on-a-chip models utilize multiple tissue lineages on a mechanically manipulated scaffold, capturing dynamic tissue–tissue interactions. Created with BioRender.com.

Transwell culturing systems maintained at ALI with proper differentiation media allow for pseudostratified epithelial growth and formation of a mucosal barrier [9,10,11,12]. Transwell systems traditionally utilize cells derived from normal tissue samples including adult basal stem cells (i.e., primary cells) [13,14,15,16,17,18] and more recently, human pluripotent stem cells (hPSC) [19,20,21,22], although hPSC systems may require longer differentiation timescales and often involve lentiviral-mediated delivery of cell reprogramming factors [19,20,21]. Primary cells can be acquired directly through epithelial brushing or tissue digestion from human and animal model sources, after which cell suspensions can be frozen down for future use or, like hPSC, expanded in normal 2D cell culture format [13,14,15,16,17,18]. Basal progenitor cells are then transferred to extracellular matrix-coated Transwell membranes and maintained in submerged conditions until the cells reach confluence. Finally, media is removed from the apical chamber and in some systems, a new media is required to maximize differentiation into a polarized epithelium over several weeks (Figure 1). Currently, several commercially available media systems and cell suppliers exist (e.g., Lonza, STEMCELL Technologies), allowing widespread access to these systems without the need for in-house media development or optimization. Transwell systems in 96-well format have also been developed, allowing for high-throughput utility [23]. Importantly, ALI cultures have been shown to recapitulate the morphology and physiology of the upper conducting airways of the normal human respiratory epithelium, including rare cell populations found in vivo [9,10,11,12]. Additionally, as models of airway disease, they can recapitulate major clinical features of cystic fibrosis (CF) [24,25,26,27,28], asthma [29,30,31,32,33], and chronic obstructive pulmonary disease (COPD) [30,34,35].

One limitation of primary cell-derived models is that differentiation capacity of these cells is lost progressively upon expansion in traditional 2D cell culture settings. Fresh primary cells have to be acquired, and cell stocks from individual donors bought commercially can be exhausted. Despite this, media supplemented with a Rho-kinase inhibitor can enhance cell proliferation and viability prior to differentiation [36,37]. Additionally, passaging these undifferentiated basal cells in the presence of irradiated fibroblasts alongside Rho-kinase inhibition leads to so-called “conditionally reprogrammed cells” which allows additional passages without loss of multipotent differentiation capacity [36,37]. These systems are not only cost-saving, but allow a larger window for genetic manipulation, selection, and cryopreservation of larger stocks. The establishment of an immortalized basal cell line (termed BSCi-NS1.1 cells) which is capable of differentiation up to passage thirty has extended this limit even further [38]. Another immortalized cell line is the well-characterized Calu-3 line which can be propagated indefinitely, but whose differentiation is limited to secretory and non-ciliated cells [39]. Still, primary cell-derived ALI cultures have proven to be genetically tractable systems for knockdown (e.g., shRNA) or knockout (e.g., CRISPR/Cas9 targeting) of desired genes with [40,41] or without [42] extending cellular differentiation capacity limits.

### 2.2. Organoids

More recently, in vitro organoid systems have been developed that allow recapitulation of organ development through self-organizing tissues and platforms in both liquid culture and 3D scaffolding matrices. Unlike ALI systems which have a membrane scaffold that dictates airway lumen development, organoids self-organize such that the lumen can be either external or internal (see Figure 1). These systems use hPSC [22,43,44,45,46,47,48] or primary cells derived directly from mature tissue [45,49,50,51]. Both hPSC and primary cell organoid differentiation can recapitulate the mature proximal lung [43,44,45,46,47,48,49,50]. As with ALI systems, hPSC-derived organoids can require longer differentiation timescales [43,44] and can result in matching in utero tissue development time, particularly for distal airway structures [52,53]. Because of this, one hurdle of hPSC systems for lung organoid development is recapitulation of the lower lung [52,53,54,55,56,57]. For instance, while alveolar cell markers can be detected in a subpopulation of cells, these appear to represent bipotential progenitor cells [52,56]. Nevertheless, general progress has been made recently (reviewed here: [54,55]), in particular for primary cell organoid systems [58,59].

As organoid progenitor cells can maintain differentiation capacity after cryostorage [49], they offer the possibility of perpetual cell culture expansion. This allows for banked donor material to be compared in multiple laboratories and, in the case of primary cells, against past and future donor stocks [49]. Additionally, these systems are proving tractable to genetic manipulations such as knock-in [60] and lentiviral transduction [45], and can be grown in a multiwell format allowing for high-throughput screening of host tissue response to a multitude of signaling or environmental factors [61,62].

### 2.3. Incorporation of Airway Mechanics, Heterotypic Cell–Cell Interactions, and On-Chip Systems

In vivo, lung cells experience dynamic forces both from the expansion and contraction of tidal breathing, as well as perfusion from extensive vasculature [63]. These mechanical stresses and their impacts on airway biology are increasingly being investigated with in vitro culture systems to better recapitulate the native airway environment. For example, Tarran et al. subjected primary human bronchial cultures at ALI to rotational shear forces to mimic the dynamic mechanical stress experienced by much of the respiratory tree during inspiration and expiration [24]. Using this system, they found that the airway surface liquid (ASL) ion imbalance found under standard conditions in cultures from CF donors could be rescued through mechanical stimulation of luminal ATP release [24]. In addition, Dimova et al. were able to stabilize primary nasal ALI Transwell cultures by using a perfusion system, thereby preventing a progressive loss of ciliated cells over time [64].

In addition to mechanical systems which recapitulate additional features of the normal human lung, co-culture of human pulmonary cells with other lineages is being actively explored. While current organoid and ALI systems better model proximal rather than distal airways, in vivo studies revealed that endothelial expression of Tsp1 is critical to alveolar lineage differentiation [65]. Lee et al., then demonstrated that co-culture of primary murine bronchoalveolar cells and Tsp1-expressing lung endothelial cells in an in vitro organoid model led to better recapitulation of alveolar markers [65]. Barkauskas et al. found that primary human type II alveolar epithelial cells only grew into self-renewing alveolar organoids when co-cultured with a fetal human pulmonary fibroblast cell line, and that mature type II alveolar cells could be found even after subculture [66]. In a murine model of pneumonectomy, Lechner et al., demonstrated that monocytes and M2-like macrophages accumulated at sites of type II alveolar cell proliferation in vivo [67]. Extending this to a co-culture model of primary type II alveolar cells, they found that macrophages had a dose-dependent effect on the development of alveolar organoids which could be found expressing both type II and type I alveolar epithelial cell markers [67]. Together, these studies highlight the importance of heterotypic cell–cell interactions in model development.

More recently, ‘organ-on-a-chip’ models have combined co-culture systems with mechanical shear stress and microfluidics perfusion (Figure 1) [68]. The chips are small (~2 cm-long) and can feature a flexible membrane which acts as both an ALI scaffold and a simulated source of tidal breathing through vacuum-mediated expansion and contraction. These chip systems also offer the ability to model dynamic cross-talk between epithelial, endothelial [68,69], and even immune cell components [70,71,72]. Notably, endothelial-epithelial interactions can be added to in vitro systems to better recapitulate features of the distal lung [68,69,70,71], though they can also recapitulate more proximal airways as with standard ALI systems [72]. In modeling the distal human lung, barrier function was shown to be affected by simulated tidal breathing [69] and perfusion flow [71]. In particular, these chip models are promising tools for studying features of pneumonia since they can capture both epithelial–endothelial inflammatory responses [69,70,71], recruit circulating leukocytes in the perfusion chamber [70], and accommodate alveolar macrophages in the apical chamber [71]. As with classical ALI systems, perfusion chip models can also recapitulate features of chronic inflammatory diseases such as asthma [72].

## 3. Application of In Vitro Airway Models with Emergent Properties in Virology

Many studies have shown that culture systems with emergent properties recapitulate important pathological features of infection, capture host responses or viral attenuation observed in vivo, and often display phenotypes not resolvable with 2D systems. Moreover, these models are susceptible to a wide range of respiratory viruses [52,53], including difficult (e.g., rhinovirus C [73,74,75]), “unculturable” (e.g., human coronavirus HKU1 [76]; human bocavirus [77]) and novel viruses with unknown properties (e.g., SARS-CoV-2 [78]). As these systems model the natural host, and therefore viral targets for infection, their utility in studying clinical isolates is also not sensitive to ongoing viral evolution. For example, circulating influenza A virus (IAV) strains have evolved new receptor-binding properties, resulting in decreased rates of clinical sample isolation in traditional 2D cell culture systems [79,80,81,82] and rapidly selecting for artifactual in vitro mutations [80,81,82,83]. ALI systems have been shown to remove these mutations in as little as a single passage and thereby better stabilize clinical genotypes [83].

These systems also represent powerful risk assessment tools of emergent viruses, a task traditionally carried out with animal models and ex vivo tissue infections [84,85,86,87,88]. For instance, Hui et al. explored avian influenza virus replication and immune response in bronchus explant cultures alongside organoid cultures derived from the same human donor, showing that viral replication kinetics and tropism were in agreement across both systems [49]. Similarly, Zhou et al. modeled the proximal human airway through organoid development to analyze avian and swine IAV alongside 2009 pandemic H1N1, and found these organoids to be a morphologically-relevant system to assess emerging influenza viruses [50].

ALI systems are also being utilized in assessment of live attenuated viral vaccines [89,90,91,92,93,94]. The restriction of an experimental human parainfluenza virus 2 vaccine candidate in the upper respiratory tract (URT) and lower respiratory tract (LRT) of African green monkeys was recapitulated in tracheal/bronchial epithelial cultures by modulating temperature to reflect proximal or distal airways [92]. Further, the failure of the 2013–2014 US influenza season LAIV4 vaccine, which did not confer protection against circulating H1N1 to children [95,96,97,98,99], could have been predicted prior to market release through the use of ALI systems. In line with observed post-market failure, primary nasal epithelial cultures revealed an enhanced attenuated phenotype of live attenuated influenza vaccine strains not seen in 2D immortalized cell lines [89,90,91]. Similarly, an attenuated respiratory syncytial virus vaccine candidate was mildly restricted in 2D culture systems when compared with a wild-type strain, but exhibited marked restriction in replication when infecting either cotton rats or human primary cell-derived ALI cultures [93].

Beyond live attenuated vaccine candidates, ALI cultures have shown utility in screening antiviral compounds [100,101,102]. Work by Boda et al. used this model system as a platform to screen inhibitors of rhinovirus and influenza virus replication [100]. An inhibitor of influenza virus replication in ALI cultures was also shown to be protective against severe disease in ferrets [101] and more recently, alongside remdesivir, was shown to broadly inhibit emergent coronaviruses, including SARS-CoV-2 [102].

Meaningful human- and donor-specific data can be captured through emergent culture systems in lieu of challenge experiments, which are hindered by the severity of the agent being tested and participant acquisition [103,104]. For example, work by Peretz et al. showed that cultures responded to sex hormones according to the sex of the primary cell donor. Specifically, female—but not male—donors restricted IAV replication after exposure to estrogenic compounds [105], providing a means to mechanistically investigate the impact sex has on airway diseases [106]. Huang et al. showed that bronchial and nasal epithelial cell ALI cultures responded to IAV infection differently according to patient-related clinical specifics [107]. Similarly, work by Honce et al. found that bronchial epithelial cell cultures derived from obese donors had reduced interferon responses and increased viral replication [108]. In an ‘asthma-on-a-chip’ format, Nawroth et al., found that while exogenous interleukin 13 recapitulated hallmarks of human asthma (e.g., goblet cell hyperplasia), rhinovirus replication was unaffected, in part due to altered antiviral and chemokine secretion profiles [72].

More generally, these systems have been widely used to assess viral cellular tropism [49,76,109,110,111,112], apical or basolateral sites of viral entry and release [53,76,109,113], and component cell type-specific responses to viral infection [71,72]. ALI Transwell models are also increasingly genetically tractable, further enabling the mechanistic dissection of specific host factors with regard to infection phenotypes. While examples of this are currently scarce, knockout of the rhinovirus C receptor cadherin related family member 3 (CDHR3) in a polarized model of airway epithelium was recently achieved to gain insight into CDHR3 function and impact on rhinovirus C infection [41].

## 4. Components of the Extracellular Barrier in the Respiratory Tract

As described above, in vitro models of the respiratory tract that recapitulate various aspects of the native microenvironment have been widely utilized to gain novel insight into virus–host interactions. Importantly, infection of the respiratory tract in vivo requires viruses to successfully penetrate the mucus barrier and work to date has highlighted both pro- and antiviral roles of barrier components (e.g., mucins [114,115], defensins [116,117,118,119], proteases [120,121,122,123,124,125,126,127,128,129,130,131,132,133,134,135,136,137,138,139], antiproteases [123,140], lactoferrin [141], exosomes [142], and microbiota [143]). 3D models attempt to recreate the respiratory tract mucosal barrier in vitro, and therefore must incorporate important components of the in vivo barrier, some of which are defined in greater detail below.

### 4.1. Mucus and Mucins

**Figure 2:**
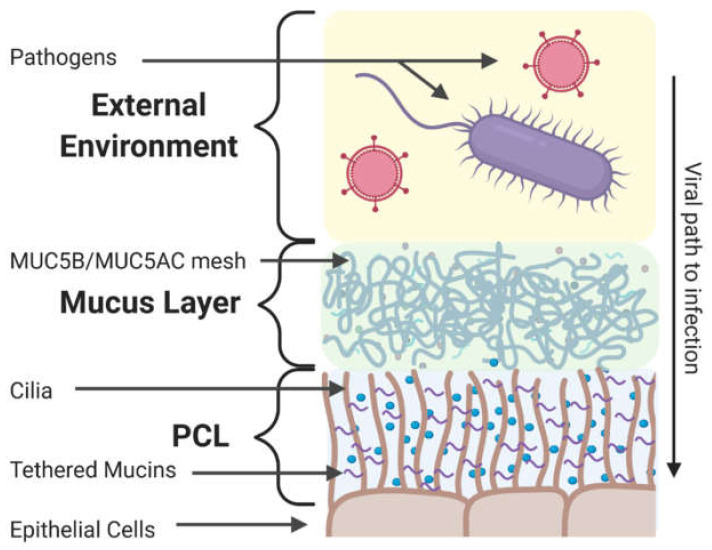
Cross-section of the respiratory tract mucosal barrier. Epithelial cells of the proximal airways are protected by a dense PCL and overlying mucus gel layer. Pathogens can be slowed or trapped in this restrictive gel while the coordinated beating of underlying ciliated epithelial cells propels this gel away from the more vulnerable distal airways towards gastrointestinal clearance. Created with BioRender.com.

Similar to other mucosal tissues, the extracellular barrier in the respiratory tract consists of a secreted mucus gel and an underlying periciliary layer (PCL) that provides structural stability to the mucus layer [144] as depicted in Figure 2. The physicochemical properties of mucus such as viscosity, pH, and thickness (~10–50 µm) [145,146] are mediated by ions [28,147,148,149]. While fluctuating in various diseased states [150], the pH of respiratory mucus is normally slightly acidic (~6.6) with alkinalization facilitating the formation of the mucus gel [151]. The mucus gel and PCL contain mucin glycoproteins which organize into a porous meshwork and fall into three categories: secreted polymerizing mucins, secreted non-polymerizing mucins, and membrane-associated tethered mucins.

The mucus gel of the respiratory tract mainly consists of the secreted polymeric mucins MUC5B and MUC5AC. While these mucins form the mucus matrix together through disulfide cross-linking and/or physical entanglements, MUC5B and MUC5AC are differentially expressed [152,153,154] and structured, and serve different roles in mucosal immunity [114,155,156,157,158,159,160,161]. Notably, the specific roles MUC5B and MUC5C play in viral neutralization are still unclear. MUC5B is most abundantly expressed in submucosal glands and epithelium along the respiratory tract while MUC5AC is primarily found in the nasal respiratory epithelium and segmental bronchi [153,154]. Both mucins are absent from the terminal bronchioles [152,153], which correlates with the mucus layer gradient (thick in the URT to absent in the LRT) [152,153]. MUC5B, but not MUC5AC, is essential for proper mucociliary clearance (MCC) [155]—the primary method by which mucus (and any trapped pathogens) is removed from the lung by the coordinated beating of cilia within the PCL. Either a lack or overexpression of MUC5B leads to defective MCC by changing viscosity and disrupting mucus equilibrium [155,156,157].

While the vast majority of secreted mucins polymerize into gels, MUC7 and MUC8 are soluble and do not polymerize. There is little literature on MUC7 in the respiratory tract, perhaps reflecting the fact MUC7 expression appears to be limited to submucosal glands (and occasionally goblet cells) at very low expression levels [162,163]. The functions and characteristics of MUC8 are also poorly understood, in part because the cDNA sequence and the murine homolog have not yet been determined [164]. In one study, MUC8 siRNA silencing resulted in altered cytokine production, indicating that MUC8 may function as an anti-inflammatory mucin [165].

Tethered mucins are found in the PCL where they contribute to airway surface hydration and create a mesh denser than that of the mucus gel [144]. Tethered mucins lack the cytosine-rich termini characteristic of secreted mucins. Instead, they are anchored to the cell surface by a transmembrane domain downstream of their heavily glycosylated, “brush-like” extracellular domain, and have a short cytosolic tail which enables interaction with cell signaling pathways [166,167,168,169]. Beyond their ability to form a physical barrier, shedding of the extracellular domain from the epithelial surface [9,166,170] may facilitate viral clearance [115]. Notably, although tethered mucins are expressed on epithelial cells throughout the respiratory tract, they are localized to specific sites within the microenvironment (MUC1, microvilli; MUC4 and MUC20, cilia; MUC16, goblet cells) [9,171]. This distribution, along with the diverse array of carbohydrates linked to their extracellular protein cores and differences in cytoplasmic tail sequence, may underlie mucin-specific effects in viral infection [9,166,172,173].

### 4.2. Secreted (Non-Mucin) Components

Secreted proteins such as defensins, proteases, immunoglobulins, and extracellular vesicles such as exosomes provide mucus with additional physicochemical and immunological properties, and greatly influence viral invasion and pathogenesis. Growth factors, transferrins such as lactoferrin, and other immune cells also found in the respiratory tract are not covered in this review.

#### 4.2.1. Defensins

Defensins are antimicrobial peptides which neutralize bacteria, viruses, and fungi by attracting immune cells and directly interacting with pathogens. Defensins are categorized alpha, beta, or theta based on their chemical structure, but all are small (18-45 residues), cationic, amphipathic, and have β-sheet regions [116]. α-defensins are further divided into myeloid (intracellular action) and enteric (extracellular action) [116]. While enteric α-defensins are absent from the respiratory tract, myeloid (neutrophilic) defensin expression increases in the respiratory tract during viral infection [174,175]. β-defensins are ubiquitously expressed by epithelial cells throughout the body, but β-defensin-1, -2, and -3 are the most studied respiratory tract β-defensins [116,176]. In addition to their antimicrobial role, β-defensins have been found to serve as both proinflammatory mediators [177,178,179] and attenuators [180,181,182] of the innate immune response, with the former better understood than the latter [183]. θ-defensins are not naturally expressed in humans and only exist in our genome as pseudogenes [116]. However, synthetic peptides (retrocyclins) partially derived from these human θ-defensin pseudogenes have antiviral potential against human immunodeficiency virus, herpes simplex virus, and IAV in vitro [117,184].

#### 4.2.2. Proteases

Unchecked proteolytic activity can lead to tissue damage and chronic lung disease [185,186,187], highlighting the importance of protease/antiprotease balance to maintaining lung health [188]. This balance also can influence susceptibility to viral infection as proteases tend to act as activators of viral glycoproteins [123]. Common proteases of the respiratory tract include type II transmembrane serine proteases, cysteine proteases, and matrix metalloproteinases, the latter of which contribute to the pathogenesis of various lung diseases (cysteine proteases [189,190,191,192,193,194,195,196,197]; matrix metalloproteinases [198,199,200,201,202]).

The type II transmembrane serine protease family is characterized by a nucleophilic serine and includes human airway trypsin-like protease (HAT), matriptase, and hespin/transmembrane protease serine 2 (TMPRSS2). Notably, HAT upregulates MUC5AC [203], thereby increasing mucus production, and matriptase degrades the extracellular matrix which can lead to tissue alterations [204]. Additionally, TMPRSS2 activates many major respiratory viruses [123] including IAV [124,125,126,133,134,136] and coronaviruses (SARS-CoV, SARS-CoV-2) [121,127,128,130]. Cysteine proteases, a subfamily of cathepsins, cleave with a cysteine residue as the nucleophile target and include cathepsin B, K, L, and S in the respiratory tract [123]. Under normal conditions, cysteine proteases provide many important immune functions including inflammation control, macrophage function, class II-associated Ii peptide degradation in major histocompatibility complex class II molecules, generation of CD4+ T cells, and toll-like receptor-9 signaling [205,206,207,208,209,210]. However, cysteine proteases can cause serious damage to the extracellular matrix and tissues [185,186,187] when activated by acidic conditions [211,212]. Matrix metalloproteinases are zinc-dependent endopeptidases which primarily degrade extracellular matrix components, but also activate/deactivate signaling proteins such as cytokines. Due to their highly-degradative nature, matrix metalloproteinases are found in low concentrations in the healthy adult lung and expression is tightly regulated [213]. However, matrix metalloproteinases play essential roles in fetal lung development [214,215], and epithelial cell migration and tissue remodeling during lung wound repair [216,217,218,219,220].

#### 4.2.3. Other Secreted Components

Exosomes, a form of extracellular vesicle, play an important role in cell–cell communication and mucosal barrier immunity via transportation of a wide variety of cargo including proteins, lipids, DNA, and RNA [142,221,222,223,224]. Exosomes may block viral infection through direct interactions [142] while various other publications have highlighted the role of exosomes in the lung immune response, both innate and adaptive, and pro- and anti-inflammatory [225,226,227,228,229]. Considerable quantities of secretory immunoglobulins (IgG and IgA) are also located in respiratory mucus, and serve to neutralize foreign antigens captured by the mucosal barrier [230,231]. In addition to their immunological role, immunoglobulin expression has been found to impact mucus secretion; approximately 44% of patients with humoral primary immunodeficiencies (low levels of IgG, IgA, and IgM) suffer from mucus plugging, where high quantities of mucus block the respiratory tract [232].

### 4.3. Microbiota

The respiratory tract microbiota decreases in biomass from URT to LRT as exposure to the environment decreases [233]. Each niche along the URT (e.g., nasal cavity, nasopharynx, sinuses, oral cavity, oropharynx) has its own distinct microbiota [234]. While the microbial population found at any one time varies widely from person to person, studies suggest the existence of an underlying core URT microbiota shared between the majority of healthy individuals in the same population [235,236,237]. The LRT, traditionally considered sterile, has a diverse collection of bacteria resident to both healthy and diseased respiratory tracts. While the most common method of analyzing LRT microbiota involves passing a bronchoscope through the URT, studies have confirmed the presence of a LRT microbiota and concluded that microbiota found in bronchoalveolar lavage samples were not exclusively a result of URT bronchoscope contamination [238,239]. However, there is little evidence supporting the existence of a common LRT microbiota amongst healthy individuals [233] and multiple studies have proposed that microbes migrate from URT to LRT through passive microaspiration [240,241,242]. Overall, while the bacterial load in the lung is low compared to other tissues (e.g., the gastrointestinal tract), multiple studies have detected a connection between niche respiratory microbiota populations and innate defenses against respiratory viruses such as respiratory syncytial virus, rhinovirus, human metapneumovirus, and IAV in vivo in infants [243,244,245], children [246], adults [247], and mouse models [143].

## 5. Utility of In Vitro Airway Models with Emergent Properties in Probing Virus Interactions in the Extracellular Space

As noted in the Introduction, cell lines and animal models fail to recapitulate critical aspects of the human airway microenvironment not only at the intracellular, but also the extracellular level. Specifically, cell lines lack a complete representation of the polarized glycocalyx and mucosal barrier [248,249] while animal models exhibit species-specific differences (e.g., in mucin structure [250], mucin-associated glycans [251,252]). Thus, in vitro models with emergent properties may also be useful tools for many questions underlying airway infection biology at the mucosal surface.

Organoids, for instance, recapitulate epithelial cell diversity of the proximal airways [43,44,45,46,47,48,49,50], components of the PCL [52,253], and secrete gel-forming mucins [43,45,49,50,52] as well as other factors of the ASL [254]. However, while not always the case [50], airway organoids can develop with the apical lumen internal to their spherical structure (see Figure 1) [45,47,49,50], posing a problem for viruses which require access to the apical epithelial surface. Several approaches have been taken to overcome this problem such as microinjection [53], shearing by pasteur pipettes [49], or enzymatic dissociation and culturing on a 2D format [50], though the latter can impact the pseudostratified nature of the culture. In these apical-internal organoid systems [45,47,49,50], both access to and analysis of the mucus gel is very difficult. Additionally, a totally enclosed apical surface prevents normal MCC, as all apical secretions—except in cases of transcytosis across the epithelial barrier—are recalcitrant.

Conversely, when the apical lumen is external to the organoid, secretory components intermix with organoid matrix and media components. This dilution may prevent formation of any normal mucus gel layer and impact the architecture of the PCL. Overall, little investigation has been done on the degree to which this system recapitulates the secretory components of the human airways and organoids have not yet been utilized effectively for virus-mucus interaction studies.

In contrast to organoid cultures, differentiation of cells at ALI in Transwell models enables easy access to both the apical and basolateral components of the same culture (Figure 1) and, perhaps as a result, ALI systems have been widely investigated for their PCL and mucus gel properties. For instance, the well-developed PCL of primary cell-derived ALI cultures has been shown to be capable of frustrating viral access to the apical membrane [255] while the secreted mucin gel can entrap virions [256]. Vahey and Fletcher utilized the immortalized and Transwell ALI-differentiable Calu-3 line to demonstrate that influenza virion pleomorphy allowed for specific glycoprotein orientation and enhanced penetration through this protective gel [257]. Moreover, the composition of the mucus gel in nasal and bronchial epithelial cell-derived culture systems is very similar to ex vivo endotracheal tube-derived mucus samples [258]. Mucus samples collected from ALI cultures also show comparable microstructural properties as those collected ex vivo [258,259]; still, the extent to which ALI culture-generated mucus phenocopies native or diseased mucus is not completely understood.

Interestingly, barrier properties of diseased-state mucus are thought to be enhanced as a result of increased concentration of mucins [260,261,262] and oxidative state changes [260], both of which contribute to a higher degree of mucin cross-linking [260] and therefore reduced gel pore sizes [263]. However, these reinforced barrier properties fail to explain similar susceptibility to viral infections by individuals with diseased mucus states [264,265]. One way to explore the barrier properties of healthy and diseased mucus is through the use of clinical samples [261,262,266,267]. Normal healthy human mucus can be collected through induction by nebulized hypertonic saline inhalation and subsequent tracheal aspiration [261,267], or collected from endotracheal tubes [258], although the former can impact hydration and therefore percent solid content of the mucus gel. Patients with CF [26,266] or COPD [262,268] can spontaneously expectorate enough mucus for rheological or other analysis. The differentiation of cells from diseased lungs also provides an opportunity to assay infection in cultures with altered barrier states. Indeed, viral spread was observed to be restricted in CF (versus normal) ALI cultures following human parainfluenza virus 3 infection [109]. Alternatively, ALI cultures can be used to explore this disconnect as cessation of regular washing with saline leads to dramatic accumulation of mucus and total solids concentration in excess of values observed in patients with obstructive mucus diseases [262]. The addition of nebulized or liquid saline then allows for tunable concentration and hydration of ALI mucus gels [267]. Overall, the ALI format is readily amenable to viral infection (see Section 3) and culture-state measurements before and after viral infection, such as oxidative level [260], ASL height, cilia beat frequency, and MCC, in both normal and diseased proximal airway contexts (see Section 6) [24,25,26,28,269].

## 6. Assaying Virus–Host Interactions in the Extracellular Space In Vitro

### 6.1. Host-Specific Barrier Properties and How to Assess Them

The mucosal layer that coats the airway epithelium acts as a physical and chemical barrier against inhaled particulates and pathogens [270,271]. Viral infection and spread within the respiratory tract is influenced by the content and properties of the mucosal barrier. Conversely, viral infection may alter these properties and impact lung function. Thus, tools that can be applied to define barrier properties in in vitro systems are essential.

As reviewed in Section 4 and depicted in Figure 2, secreted mucins polymerize through cross-linking and physical entanglements, creating a porous microstructure which controls the transport of nano- and microscale entities, such as viruses [270,272]. Microstructural pore size can be measured through particle tracking microrheology [259,273]. For instance, pore size is measured using the trajectory of fluorescent nanoparticles with muco-inert surface chemistry, as their diffusion is mediated solely by steric obstruction from the mucus mesh [259,266,273,274]. Direct microrheological analysis of ALI culture mucus, as opposed to collection through culture washing and subsequent concentration and dialysis, is possible [275]; however, this approach requires cilia immobilization to prevent drift of probe particles. The concentration of individual mucins can be readily determined through relative (e.g., Western blot [276]) and absolute (e.g., enzyme-linked immunosorbent assay [277]) assays, though hyperaccumulation of mucus can interfere with immunological readouts and necessitate quantitation through chromatography [26,268].

Dehydration or excessive osmotic pressure from mucus hyperaccumulation, as seen in CF patient-derived ALI cultures, can lead to a collapse of the culture PCL and a cessation of MCC [267]. Additionally, CF ALI cultures experience ASL dysfunction after viral perturbation, potentially enhancing subsequent mucoadhesion and bacterial infection risk [24] observed in clinical CF exacerbations after viral respiratory infection [264]. PCL height of ALI cultures can be measured by adding differentially-sized fluorescent polymers or nanoparticles and visualized through confocal microscopy where the PCL is located within the non-overlapping regions as a result of size exclusion of larger particles [144]. Compromised PCL height negatively impacts MCC [267] which is associated with increased susceptibility to respiratory infections in vivo [264].

**Figure 3:**
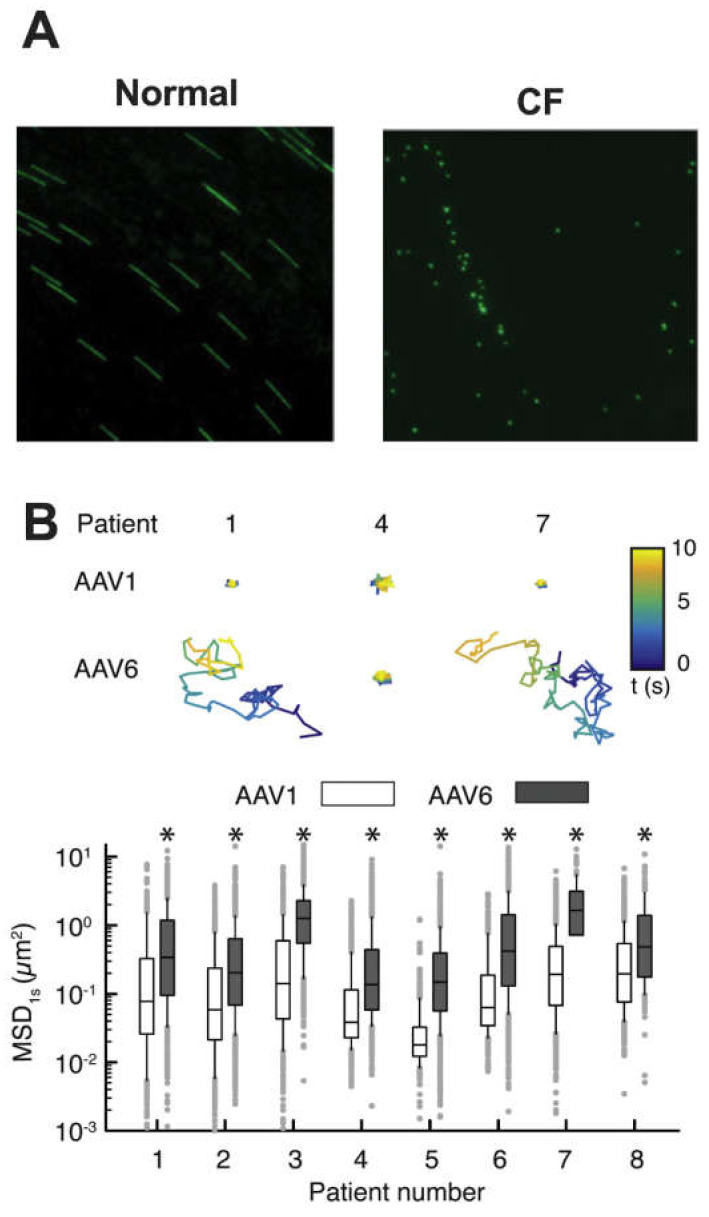
Probing the extracellular barrier: (**A**) Representative 2 s exposure image of fluorescent microspheres to illustrate mucociliary transport on normal and CF human tracheobronchial epithelial cell cultures. Reprinted with permission from Button, B.; Picher, M.; Boucher, R. “Differential effects of cyclic and constant stress on ATP release and mucociliary transport by human airway epithelia”. The Journal of Physiology 2007, 580(2), 577-592. Copyright 2007, The Physiological Society [285]. (**B**) Trajectories and mean squared displacement (MSD) of adeno-associated virus serotype 1 (AAV1) and adeno-associated virus serotype 6 (AAV6) in sputum samples from CF patients, * *p* < 0.05; Mann-Whitney test. Reprinted with permission from Duncan, G.; Kim, N.; Colon-Cortes, Y.; Rodriguez, J.; Mazur, M.; Birket, S.; Rowe, S.; West, N.; Livraghi-Butrico, A.; Boucher, R.; Hanes, J.; Aslanidi, G.; and Suk, J. “An adeno-associated viral vector capable of penetrating the mucus barrier to inhaled gene therapy” (https://doi.org/10.1016/j.omtm.2018.03.006). Molecular Therapy—Methods & Clinical Development 2018, 9:296-304. Copyright 2018, The American Society of Gene and Cell Therapy [286].

Ciliary action and mucus rheological properties give rise to MCC [278,279,280]. Some viral infections have been associated with reduced cilia beat frequency [269,281]; therefore, measuring cilia beat frequency is also important to understanding host barrier function and its viral interactions. Microscopic high-speed video of ciliary action can lead to determination of cilia beat frequency of a given region [278,282] and even the directionality of ciliary strokes [283]. Notably, higher cilia beat frequency does not necessarily correlate with increased MCC [284] as the rheological properties of mucus also impact MCC [278,279]. Mucus layer velocity is calculated by tracking large (e.g., 2 µm) fluorescent particles entrapped in the mucus network, and long-exposure images depict the path flow (see Figure 3A) [280,285].

### 6.2. Viral Particle Tracking, Host–Virus Interactions, and Specific Barrier Component Contributions

Viral transit through the mucus gel and subsequent PCL is a necessary component of all respiratory infections (see Section 4), and therefore evaluating the diffusion of viral particles through mucus represents an important aspect of viral pathogenesis. Individual virions can be tracked in real time by directly labelling viral particles with reactive, lipophilic, or intercalating dyes [287]. Quantum dots, a type of semiconductor nanoparticles, can also be used to label virions [288] without significantly impacting infectivity [289]. Once labeled, particles can be imaged directly [290] in mucus or engineered surrogates [273]. Trajectories of virion movement can be imaged, as shown in Figure 3B, to measure diffusion and mucus penetration [272,286].

As opposed to muco-inert particles used to study microrheology, viral particles often exhibit adhesive interactions with airway mucus components [286]. The measured pore sizes of airway mucus (~200–500 nm) would imply rapid diffusion of viral particles through the mucus layer based on viral particle size [259,266]. However, adhesive interactions between viral surface glycoprotein domains have been shown to significantly reduce viral diffusion through airway mucus [257,291]. For example, particle tracking microrheology studies using fluorescently-labelled adeno-associated virus revealed that diffusion of the 20 nm virions through CF sputum was substantially slower compared to 100 nm nanoparticles, which are significantly larger [292]. Importantly, viral particle tracking can be done with any mucus source, including directly on ALI systems. Evidence of viral adhesion can then be further investigated outside the context of 3D model systems using surface plasmon resonance [293], optical tweezers and atomic force microscopy [294], or a quartz crystal microbalance [295]. However, to date there have been few attempts at direct tracking of viral particles in mucus gel or on ALI systems [286].

Finally, engineered mucus hydrogels and genetic ablation of mucin expression in ALI or organoid systems represent potentially powerful tools to study the contributions of specific barrier components to infection. Engineered mucus can be produced in large volumes and can be tuned to desired parameters [273,296,297,298] such as variable cross-linking concentration [296] or mucin gels composed of only MUC5B or MUC5AC [273,297]. As with ex vivo mucus, these surrogate mucin gels could then be applied to in vitro systems to explore infection phenotypes. However, difficulty in mimicking both bulk and microrheological properties of native mucus combined with the genetic tractability of in vitro culture systems (see Section 2) highlights the utility in creating modified mucus gels through altered gene expression within the context of in vitro human ASL. Similarly, the contribution of tethered mucins as well as other host factors in the ASL can be dissected at baseline and during viral infection. For instance, CRISPR/Cas9-mediated depletion of the tethered mucin MUC18 from ALI cultures suggests a general pro-inflammatory role [40]. Koh et al. demonstrated that ablation of the SAM-pointed domain containing ETS transcription factor (SPDEF) from ALI cultures prevented MUC5AC induction and subsequent MCC impairment after stimulation with interleukin 13 [42].

Still, more work remains to dissect the contribution that individual mucins and other respiratory factors make towards a functional ASL barrier which protects from viral infection. Additionally, the extent to which individual host factors influence viral pathogenesis in both healthy and diseased human airways still needs to be addressed.

## 7. Conclusions and Future Perspectives

Understanding mucosal barriers is paramount in virology given the number of important pathogens (coronaviruses, adenovirus, human immunodeficiency virus, human papillomavirus, influenza virus, rhinovirus, and so forth) that infect via this route. To identify specific components of these barrier tissues that influence infection, researchers have utilized a variety of model systems ranging from cell lines to whole organisms. While no model system is without drawbacks and limitations, 3D in vitro models offer the benefit of mirroring the in vivo mucosal microenvironment while providing the opportunity for detailed mechanistic studies on virus–host interactions. Further, ex vivo patient-derived samples can be used to directly interrogate the barrier function of human mucus and may help, in part, to bridge the gap between 3D in vitro systems and the respiratory tract in vivo.

A better understanding of virus-mucus interactions may provide new insight into the onset and severity of viral infection. In order to initiate infection, viruses deposited on the airway surface must overcome the mucus barrier to avoid removal via MCC. Some have argued that reduced mucus clearance explains the susceptibility of individuals with chronic lung disease (e.g., COPD, asthma) to exacerbation-inducing respiratory virus infections [265]. However, individuals with chronic lung disease often possess alterations to the mucosal barrier (e.g., increased mucus gel concentration and thickness) that would seem to reinforce barrier function. Furthermore, the underlying PCL presents an additional physical barrier to infection with the tethered mucin mesh possessing network sizes less than 40 nm. Thus, the mechanism by which viruses with diameters 2–3 times larger than this (e.g., influenza virus, SARS-CoV-2) can bypass the PCL to infect ciliated cells is unclear. The model systems, in combination with the assays, described in this review may provide the means to address these and other long-standing questions on respiratory viral infectious diseases.

The state-of-the-art 3D in vitro systems discussed in this review have proven useful and will continue to be essential for studies on viral infections in mucosal tissues. However, incorporating additional components of the respiratory tract should be considered to address their limitations. As highlighted here and elsewhere, the interaction between respiratory tract microbiota, the immune response, and viral pathogens has been demonstrated in a wide variety of studies. However, immune cells and microbiota remain absent from almost all 3D models of respiratory viral infection. The lack of microbiota in 3D models does mark a significant difference between respiratory infection in vivo versus in vitro and the impact of said difference on in vitro experimental results of viral infection are unknown. The impact of MCC on infection has been challenging to re-create in 3D in vitro systems as they are typically designed as a closed system with no point of exit for viruses entrapped within the mucus layer. To include this important innate defense mechanism, adaptations to the lung-on-a-chip systems could be made that allow for MCC-mediated elimination of viral pathogens. When viral infections are initiated in vitro, the mucosal layer is often removed by washing and aspiration prior to infection. The administration of viruses to cultures with the mucus barrier intact would be ideal in order to mimic natural infections. In addition, the delivery of viruses in aerosol to 3D in vitro systems would more closely mimic the inhaled transmission route. This could be achieved using commercially available in vitro aerosol exposure systems (e.g., VITROCELL^®^ Cloud Systems). Including these components into existing platforms will significantly improve our ability to model and study critical facets of host defenses against respiratory viruses.

## Abbreviations

AAV1	adeno-associated virus serotype 1
AAV6	adeno-associated virus serotype 6
ALI	air–liquid interface
ASL	airway surface liquid
CF	cystic fibrosis
CDHR3	cadherin related family member 3
COPD	chronic obstructive pulmonary disorder
HAT	human airway trypsin-like protease
hPSC	human pluripotent stem cell
IAV	influenza A virus
LRT	lower respiratory tract
MCC	mucociliary clearance
MSD	mean squared displacement
PCL	periciliary layer
SARS-CoV-2	severe acute respiratory syndrome coronavirus 2
TMPRSS2	transmembrane protease serine 2
URT	upper respiratory tract

## References

[1] Lechner J.F., Haugen A., McClendon I.A., Pettis E.W. (1982). Clonal growth of normal adult human bronchial epithelial cells in a serum-free medium. In Vitro.

[2] Jorissen M., Van der Schueren B., Van den Berghe H., Cassiman J.J. (1989). The preservation and regeneration of cilia on human nasal epithelial cells cultured in vitro. Arch. Otorhinolaryngol..

[3] Wu R., Yankaskas J., Cheng E., Knowles M.R., Boucher R. (1985). Growth and differentiation of human nasal epithelial cells in culture. Serum-free, hormone-supplemented medium and proteoglycan synthesis. Am. Rev. Respir. Dis..

[4] Benali R., Tournier J.M., Chevillard M., Zahm J.M., Klossek J.M., Hinnrasky J., Gaillard D., Maquart F.X., Puchelle E. (1993). Tubule formation by human surface respiratory epithelial cells cultured in a three-dimensional collagen lattice. Am. J. Physiol..

[5] Whitcutt M.J., Adler K.B., Wu R. (1988). A biphasic chamber system for maintaining polarity of differentiation of cultured respiratory tract epithelial cells. In Vitro Cell. Dev. Biol..

[6] Wheeler A.H., Nungester W.J. (1942). Effect of mucin on influenza virus infection in hamsters. Science.

[7] Fazekas de S Groth S. (1952). Nasal mucus and influenza viruses. I. The haemagglutinin inhibitor in nasal secretions. J. Hyg..

[8] Pannu J.S., Sigel M.M. (1963). Inhibition of viruses by secretions from the female genital tract. Proc. Soc. Exp. Biol. Med..

[9] Kesimer M., Ehre C., Burns K.A., Davis C.W., Sheehan J.K., Pickles R.J. (2013). Molecular organization of the mucins and glycocalyx underlying mucus transport over mucosal surfaces of the airways. Mucosal Immunol..

[10] Davis A.S., Chertow D.S., Moyer J.E., Suzich J., Sandouk A., Dorward D.W., Logun C., Shelhamer J.H., Taubenberger J.K. (2015). Validation of normal human bronchial epithelial cells as a model for influenza A infections in human distal trachea. J. Histochem. Cytochem..

[11] Montoro D.T., Haber A.L., Biton M., Vinarsky V., Lin B., Birket S.E., Yuan F., Chen S., Leung H.M., Villoria J. (2018). A revised airway epithelial hierarchy includes CFTR-expressing ionocytes. Nature.

[12] Kesimer M., Kirkham S., Pickles R.J., Henderson A.G., Alexis N.E., Demaria G., Knight D., Thornton D.J., Sheehan J.K. (2009). Tracheobronchial air-liquid interface cell culture: A model for innate mucosal defense of the upper airways?. Am. J. Physiol. Lung Cell. Mol. Physiol..

[13] Zeng H., Goldsmith C.S., Maines T.R., Belser J.A., Gustin K.M., Pekosz A., Zaki S.R., Katz J.M., Tumpey T.M. (2013). Tropism and infectivity of influenza virus, including highly pathogenic avian H5N1 virus, in ferret tracheal differentiated primary epithelial cell cultures. J. Virol..

[14] Newby C.M., Rowe R.K., Pekosz A. (2006). Influenza A virus infection of primary differentiated airway epithelial cell cultures derived from Syrian golden hamsters. Virology.

[15] You Y., Richer E.J., Huang T., Brody S.L. (2002). Growth and differentiation of mouse tracheal epithelial cells: Selection of a proliferative population. Am. J. Physiol. Lung Cell. Mol. Physiol..

[16] Kondo M., Tamaoki J., Takeyama K., Nakata J., Nagai A. (2002). Interleukin-13 induces goblet cell differentiation in primary cell culture from Guinea pig tracheal epithelium. Am. J. Respir. Cell Mol. Biol..

[17] Fulcher M.L., Gabriel S., Burns K.A., Yankaskas J.R., Randell S.H. (2005). Well-differentiated human airway epithelial cell cultures. Methods Mol. Med..

[18] Karp P.H., Moninger T.O., Weber S.P., Nesselhauf T.S., Launspach J.L., Zabner J., Welsh M.J. (2002). An in vitro model of differentiated human airway epithelia. Methods for establishing primary cultures. Methods Mol. Biol..

[19] Firth A.L., Dargitz C.T., Qualls S.J., Menon T., Wright R., Singer O., Gage F.H., Khanna A., Verma I.M. (2014). Generation of multiciliated cells in functional airway epithelia from human induced pluripotent stem cells. Proc. Natl. Acad. Sci. USA.

[20] Firth A.L., Menon T., Parker G.S., Qualls S.J., Lewis B.M., Ke E., Dargitz C.T., Wright R., Khanna A., Gage F.H. (2015). Functional gene correction for cystic fibrosis in lung epithelial cells generated from patient iPSCs. Cell Rep..

[21] Wong A.P., Bear C.E., Chin S., Pasceri P., Thompson T.O., Huan L.-J., Ratjen F., Ellis J., Rossant J. (2012). Directed differentiation of human pluripotent stem cells into mature airway epithelia expressing functional CFTR protein. Nat. Biotechnol..

[22] Hawkins F.J., Suzuki S., Beermann M.L., Barillà C., Wang R., Villacorta-Martin C., Berical A., Jean J.C., Le Suer J., Matte T. (2020). Derivation of airway basal stem cells from human pluripotent stem cells. Cell Stem Cell.

[23] Bluhmki T., Bitzer S., Gindele J.A., Schruf E., Kiechle T., Webster M., Schymeinsky J., Ries R., Gantner F., Bischoff D. (2020). Development of a miniaturized 96-Transwell air-liquid interface human small airway epithelial model. Sci. Rep..

[24] Tarran R., Button B., Picher M., Paradiso A.M., Ribeiro C.M., Lazarowski E.R., Zhang L., Collins P.L., Pickles R.J., Fredberg J.J. (2005). Normal and cystic fibrosis airway surface liquid homeostasis. The effects of phasic shear stress and viral infections. J. Biol. Chem..

[25] Derichs N., Jin B.J., Song Y., Finkbeiner W.E., Verkman A.S. (2011). Hyperviscous airway periciliary and mucous liquid layers in cystic fibrosis measured by confocal fluorescence photobleaching. FASEB J..

[26] Henderson A.G., Ehre C., Button B., Abdullah L.H., Cai L.-H., Leigh M.W., DeMaria G.C., Matsui H., Donaldson S.H., Davis C.W. (2014). Cystic fibrosis airway secretions exhibit mucin hyperconcentration and increased osmotic pressure. J. Clin. Investig..

[27] Farberman M.M., Ibricevic A., Joseph T.D., Akers K.T., Garcia-Medina R., Crosby S., Clarke L.L., Brody S.L., Ferkol T.W. (2011). Effect of polarized release of CXC-chemokines from wild-type and cystic fibrosis murine airway epithelial cells. Am. J. Respir. Cell Mol. Biol..

[28] Tarran R., Trout L., Donaldson S.H., Boucher R.C. (2006). Soluble mediators, not cilia, determine airway surface liquid volume in normal and cystic fibrosis superficial airway epithelia. J. Gen. Physiol..

[29] Lakshmi S.P., Reddy A.T., Banno A., Reddy R.C. (2018). Airway epithelial cell peroxisome proliferator-activated receptor γ regulates inflammation and mucin expression in allergic airway disease. J. Immunol..

[30] Sotty J., Garçon G., Denayer F.O., Alleman L.Y., Saleh Y., Perdrix E., Riffault V., Dubot P., Lo-Guidice J.M., Canivet L. (2019). Toxicological effects of ambient fine (PM) and ultrafine (PM) particles in healthy and diseased 3D organo-typic mucocilary-phenotype models. Environ. Res..

[31] Stewart C.E., Torr E.E., Mohd Jamili N.H., Bosquillon C., Sayers I. (2012). Evaluation of Differentiated Human Bronchial Epithelial Cell Culture Systems for Asthma Research. J. Allergy.

[32] Parker J., Sarlang S., Thavagnanam S., Williamson G., O’donoghue D., Villenave R., Power U., Shields M., Heaney L., Skibinski G. (2010). A 3-D well-differentiated model of pediatric bronchial epithelium demonstrates unstimulated morphological differences between asthmatic and nonasthmatic cells. Pediatr. Res..

[33] Hackett T.-L., Singhera G.K., Shaheen F., Hayden P., Jackson G.R., Hegele R.G., Van Eeden S., Bai T.R., Dorscheid D.R., Knight D.A. (2011). Intrinsic phenotypic differences of asthmatic epithelium and its inflammatory responses to respiratory syncytial virus and air pollution. Am. J. Respir. Cell Mol. Biol..

[34] Leclercq B., Happillon M., Antherieu S., Hardy E.M., Alleman L.Y., Grova N., Perdrix E., Appenzeller B.M., Lo Guidice J.M., Coddeville P. (2016). Differential responses of healthy and chronic obstructive pulmonary diseased human bronchial epithelial cells repeatedly exposed to air pollution-derived PM. Environ. Pollut..

[35] Mertens T.C.J., Karmouty-Quintana H., Taube C., Hiemstra P.S. (2017). Use of airway epithelial cell culture to unravel the pathogenesis and study treatment in obstructive airway diseases. Pulm. Pharmacol. Ther..

[36] Liu X., Ory V., Chapman S., Yuan H., Albanese C., Kallakury B., Timofeeva O.A., Nealon C., Dakic A., Simic V. (2012). ROCK inhibitor and feeder cells induce the conditional reprogramming of epithelial cells. Am. J. Pathol..

[37] Suprynowicz F.A., Upadhyay G., Krawczyk E., Kramer S.C., Hebert J.D., Liu X., Yuan H., Cheluvaraju C., Clapp P.W., Boucher R.C. (2012). Conditionally reprogrammed cells represent a stem-like state of adult epithelial cells. Proc. Natl. Acad. Sci. USA.

[38] Walters M.S., Gomi K., Ashbridge B., Moore M.A.S., Arbelaez V., Heldrich J., Ding B.S., Rafii S., Staudt M.R., Crystal R.G. (2013). Generation of a human airway epithelium derived basal cell line with multipotent differentiation capacity. Respir. Res..

[39] Kreft M.E., Jerman U.D., Lasič E., Hevir-Kene N., Rižner T.L., Peternel L., Kristan K. (2015). The characterization of the human cell line Calu-3 under different culture conditions and its use as an optimized in vitro model to investigate bronchial epithelial function. Eur. J. Pharm. Sci..

[40] Chu H.W., Rios C., Huang C., Wesolowska-Andersen A., Burchard E.G., O’Connor B.P., Fingerlin T.E., Nichols D., Reynolds S.D., Seibold M.A. (2015). CRISPR-Cas9-mediated gene knockout in primary human airway epithelial cells reveals a proinflammatory role for MUC18. Gene Ther..

[41] Everman J.L., Sajuthi S., Saef B., Rios C., Stoner A.M., Numata M., Hu D., Eng C., Oh S., Rodriguez-Santana J. (2019). Functional genomics of CDHR3 confirms its role in HRV-C infection and childhood asthma exacerbations. J. Clin. Immunol..

[42] Koh K.D., Siddiqui S., Cheng D., Bonser L.R., Sun D.I., Zlock L.T., Finkbeiner W.E., Woodruff P.G., Erle D.J. (2020). Efficient RNP-directed human gene targeting reveals SPDEF is required for IL-13-induced mucostasis. Am. J. Respir. Cell Mol. Biol..

[43] Dye B.R., Dedhia P.H., Miller A.J., Nagy M.S., White E.S., Shea L.D., Spence J.R. (2016). A bioengineered niche promotes in vivo engraftment and maturation of pluripotent stem cell derived human lung organoids. eLife.

[44] Dye B.R., Hill D.R., Ferguson M.A.H., Tsai Y.H., Nagy M.S., Dyal R., Wells J.M., Mayhew C.N., Nattiv R., Klein O.D. (2015). In vitro generation of human pluripotent stem cell derived lung organoids. eLife.

[45] Sachs N., Papaspyropoulos A., Zomer-van Ommen D.D., Heo I., Böttinger L., Klay D., Weeber F., Huelsz-Prince G., Iakobachvili N., Amatngalim G.D. (2019). Long-term expanding human airway organoids for disease modeling. EMBO J..

[46] Huang S.X.L., Islam M.N., O’Neill J., Hu Z., Yang Y.-G., Chen Y.-W., Mumau M., Green M.D., Vunjak-Novakovic G., Bhattacharya J. (2014). Efficient generation of lung and airway epithelial cells from human pluripotent stem cells. Nat. Biotechnol..

[47] Konishi S., Gotoh S., Tateishi K., Yamamoto Y., Korogi Y., Nagasaki T., Matsumoto H., Muro S., Hirai T., Ito I. (2016). Directed Induction of Functional Multi-ciliated Cells in Proximal Airway Epithelial Spheroids from Human Pluripotent Stem Cells. Stem Cell Rep..

[48] McCauley K.B., Hawkins F., Serra M., Thomas D.C., Jacob A., Kotton D.N. (2017). Efficient Derivation of Functional Human Airway Epithelium from Pluripotent Stem Cells via Temporal Regulation of Wnt Signaling. Cell Stem Cell.

[49] Hui K.P.Y., Ching R.H.H., Chan S.K.H., Nicholls J.M., Sachs N., Clevers H., Peiris J.S.M., Chan M.C.W. (2018). Tropism, replication competence, and innate immune responses of influenza virus: An analysis of human airway organoids and ex-vivo bronchus cultures. Lancet Respir. Med..

[50] Zhou J., Li C., Sachs N., Chiu M.C., Wong B.H.Y., Chu H., Poon V.K.M., Wang D., Zhao X., Wen L. (2018). Differentiated human airway organoids to assess infectivity of emerging influenza virus. Proc. Natl. Acad. Sci. USA.

[51] Tan Q., Choi K.M., Sicard D., Tschumperlin D.J. (2017). Human airway organoid engineering as a step toward lung regeneration and disease modeling. Biomaterials.

[52] Chen Y.W., Huang S.X., de Carvalho A.L.R.T., Ho S.H., Islam M.N., Volpi S., Notarangelo L.D., Ciancanelli M., Casanova J.L., Bhattacharya J. (2017). A three-dimensional model of human lung development and disease from pluripotent stem cells. Nat. Cell Biol..

[53] Porotto M., Ferren M., Chen Y.W., Siu Y., Makhsous N., Rima B., Briese T., Greninger A.L., Snoeck H.W., Moscona A. (2019). Authentic modeling of human respiratory virus infection in human pluripotent stem cell-derived lung organoids. MBio.

[54] Evans K.V., Lee J.H. (2020). Alveolar wars: The rise of in vitro models to understand human lung alveolar maintenance, regeneration, and disease. Stem Cells Transl. Med..

[55] Liao D., Li H. (2020). Dissecting the niche for alveolar type II cells with alveolar organoids. Front. Cell Dev. Biol..

[56] Nikolić M.Z., Caritg O., Jeng Q., Johnson J.A., Sun D., Howell K.J., Brady J.L., Laresgoiti U., Allen G., Butler R. (2017). Human embryonic lung epithelial tips are multipotent progenitors that can be expanded in vitro as long-term self-renewing organoids. eLife.

[57] Beers M.F., Moodley Y. (2017). When is an alveolar type 2 cell an alveolar type 2 cell? A conundrum for lung stem cell biology and regenerative medicine. Am. J. Respir. Cell Mol. Biol..

[58] Katsura H., Sontake V., Tata A., Kobayashi Y., Edwards C.E., Heaton B.E., Konkimalla A., Asakura T., Mikami Y., Fritch E.J. (2020). Human lung stem cell-based alveolospheres provide insights into SARS-CoV-2 mediated interferon responses and pneumocyte dysfunction. Cell Stem Cell.

[59] Youk J., Kim T., Evans K.V., Jeong Y.I., Hur Y., Hong S.P., Kim J.H., Yi K., Kim S.Y., Na K.J. (2020). Three-dimensional human alveolar stem cell culture models reveal infection response to SARS-CoV-2. Cell Stem Cell.

[60] Yang Q., Oost K.C., Liberali P. (2020). Engineering human knock-in organoids. Nat. Cell Biol..

[61] Barkauskas C.E., Chung M.I., Fioret B., Gao X., Katsura H., Hogan B.L.M. (2017). Lung organoids: Current uses and future promise. Development.

[62] Danahay H., Pessotti A.D., Coote J., Montgomery B.E., Xia D., Wilson A., Yang H., Wang Z., Bevan L., Thomas C. (2015). Notch2 is required for inflammatory cytokine-driven goblet cell metaplasia in the lung. Cell Rep..

[63] Garcia C.S.N.B., Prota L.F.M., Morales M.M., Romero P.V., Zin W.A., Rocco P.R.M. (2006). Understanding the mechanisms of lung mechanical stress. Braz. J. Med. Biol. Res..

[64] Dimova S., Vlaeminck V., Brewster M.E., Noppe M., Jorissen M., Augustijns P. (2005). Stable ciliary activity in human nasal epithelial cells grown in a perfusion system. Int. J. Pharm..

[65] Lee J.H., Bhang D.H., Beede A., Huang T.L., Stripp B.R., Bloch K.D., Wagers A.J., Tseng Y.H., Ryeom S., Kim C.F. (2014). Lung stem cell differentiation in mice directed by endothelial cells via a BMP4-NFATc1-thrombospondin-1 axis. Cell.

[66] Barkauskas C.E., Cronce M.J., Rackley C.R., Bowie E.J., Keene D.R., Stripp B.R., Randell S.H., Noble P.W., Hogan B.L.M. (2013). Type 2 alveolar cells are stem cells in adult lung. J. Clin. Investig..

[67] Lechner A.J., Driver I.H., Lee J., Conroy C.M., Nagle A., Locksley R.M., Rock J.R. (2017). Recruited monocytes and type 2 immunity promote lung regeneration following pneumonectomy. Cell Stem Cell.

[68] Huh D., Matthews B.D., Mammoto A., Montoya-Zavala M., Hsin H.Y., Ingber D.E. (2010). Reconstituting organ-level lung functions on a chip. Science.

[69] Huh D., Leslie D.C., Matthews B.D., Fraser J.P., Jurek S., Hamilton G.A., Thorneloe K.S., McAlexander M.A., Ingber D.E. (2012). A human disease model of drug toxicity-induced pulmonary edema in a lung-on-a-chip microdevice. Sci. Transl. Med..

[70] Benam K.H., Villenave R., Lucchesi C., Varone A., Hubeau C., Lee H.H., Alves S.E., Salmon M., Ferrante T.C., Weaver J.C. (2016). Small airway-on-a-chip enables analysis of human lung inflammation and drug responses in vitro. Nat. Methods.

[71] Deinhardt-Emmer S., Rennert K., Schicke E., Cseresnyés Z., Windolph M., Nietzsche S., Heller R., Siwczak F., Haupt K.F., Carlstedt S. (2020). Co-infection with Staphylococcus aureus after primary influenza virus infection leads to damage of the endothelium in a human alveolus-on-a-chip model. Biofabrication.

[72] Nawroth J.C., Lucchesi C., Cheng D., Shukla A., Ngyuen J., Shroff T., Varone A., Karalis K., Lee H.-H., Alves S. (2020). A micro-engineered airway lung-chip models key features of viral-induced exacerbation of asthma. Am. J. Respir. Cell Mol. Biol..

[73] Hao W., Bernard K., Patel N., Ulbrandt N., Feng H., Svabek C., Wilson S., Stracener C., Wang K., Suzich J. (2012). Infection and propagation of human rhinovirus C in human airway epithelial cells. J. Virol..

[74] Ashraf S., Brockman-Schneider R., Bochkov Y.A., Pasic T.R., Gern J.E. (2013). Biological characteristics and propagation of human rhinovirus-C in differentiated sinus epithelial cells. Virology.

[75] Tapparel C., Sobo K., Constant S., Huang S., Van Belle S., Kaiser L. (2013). Growth and characterization of different human rhinovirus C types in three-dimensional human airway epithelia reconstituted in vitro. Virology.

[76] Pyrc K., Sims A.C., Dijkman R., Jebbink M., Long C., Deming D., Donaldson E., Vabret A., Baric R., van der Hoek L. (2010). Culturing the unculturable: Human coronavirus HKU1 infects, replicates, and produces progeny virions in human ciliated airway epithelial cell cultures. J. Virol..

[77] Dijkman R., Koekkoek S.M., Molenkamp R., Schildgen O., van der Hoek L. (2009). Human bocavirus can be cultured in differentiated human airway epithelial cells. J. Virol..

[78] Zhu N., Zhang D., Wang W., Li X., Yang B., Song J., Zhao X., Huang B., Shi W., Lu R. (2020). A novel coronavirus from patients with pneumonia in China, 2019. N. Engl. J. Med..

[79] Matrosovich M., Matrosovich T., Carr J., Roberts N.A., Klenk H.D. (2003). Overexpression of the alpha-2,6-sialyltransferase in MDCK cells increases influenza virus sensitivity to neuraminidase inhibitors. J. Virol..

[80] Oh D.Y., Barr I.G., Mosse J.A., Laurie K.L. (2008). MDCK-SIAT1 cells show improved isolation rates for recent human influenza viruses compared to conventional MDCK cells. J. Clin. Microbiol..

[81] Takada K., Kawakami C., Fan S., Chiba S., Zhong G., Gu C., Shimizu K., Takasaki S., Sakai-Tagawa Y., Lopes T.J.S. (2019). A humanized MDCK cell line for the efficient isolation and propagation of human influenza viruses. Nat. Microbiol..

[82] Lin Y., Wharton S.A., Whittaker L., Dai M., Ermetal B., Lo J., Pontoriero A., Baumeister E., Daniels R.S., McCauley J.W. (2017). The characteristics and antigenic properties of recently emerged subclade 3C.3a and 3C.2a human influenza A(H3N2) viruses passaged in MDCK cells. Influenza Other Respir. Viruses.

[83] Brown J.C., Barclay W.S., Galiano M., Harvey R. (2020). Passage of influenza A/H3N2 viruses in human airway cells removes artefactual variants associated with neuraminidase-mediated binding. J. Gen. Virol..

[84] Enkirch T., von Messling V. (2015). Ferret models of viral pathogenesis. Virology.

[85] Chan R.W.Y., Chan L.L.Y., Mok C.K.P., Lai J., Tao K.P., Obadan A., Chan M.C.W., Perez D.R., Peiris J.S.M., Nicholls J.M. (2017). Replication of H9 influenza viruses in the human ex vivo respiratory tract, and the influence of neuraminidase on virus release. Sci. Rep..

[86] Nicholls J.M., Chan M.C.W., Chan W.Y., Wong H.K., Cheung C.Y., Kwong D.L.W., Wong M.P., Chui W.H., Poon L.L.M., Tsao S.W. (2007). Tropism of avian influenza A (H5N1) in the upper and lower respiratory tract. Nat. Med..

[87] Weinheimer V.K., Becher A., Tönnies M., Holland G., Knepper J., Bauer T.T., Schneider P., Neudecker J., Rückert J.C., Szymanski K. (2012). Influenza A viruses target type II pneumocytes in the human lung. J. Infect. Dis..

[88] Hocke A.C., Becher A., Knepper J., Peter A., Holland G., Tönnies M., Bauer T.T., Schneider P., Neudecker J., Muth D. (2013). Emerging human middle East respiratory syndrome coronavirus causes widespread infection and alveolar damage in human lungs. Am. J. Respir. Crit. Care Med..

[89] Fischer W.A., King L.S., Lane A.P., Pekosz A. (2015). Restricted replication of the live attenuated influenza A virus vaccine during infection of primary differentiated human nasal epithelial cells. Vaccine.

[90] Fischer W.A., Chason K.D., Brighton M., Jaspers I. (2014). Live attenuated influenza vaccine strains elicit a greater innate immune response than antigenically-matched seasonal influenza viruses during infection of human nasal epithelial cell cultures. Vaccine.

[91] Wohlgemuth N., Ye Y., Fenstermacher K.J., Liu H., Lane A.P., Pekosz A. (2017). The M2 protein of live, attenuated influenza vaccine encodes a mutation that reduces replication in human nasal epithelial cells. Vaccine.

[92] Schaap-Nutt A., Scull M.A., Schmidt A.C., Murphy B.R., Pickles R.J. (2010). Growth restriction of an experimental live attenuated human parainfluenza virus type 2 vaccine in human ciliated airway epithelium in vitro parallels attenuation in African green monkeys. Vaccine.

[93] Rostad C.A., Stobart C.C., Gilbert B.E., Pickles R.J., Hotard A.L., Meng J., Blanco J.C.G., Moin S.M., Graham B.S., Piedra P.A. (2016). A recombinant respiratory syncytial virus vaccine candidate attenuated by a low-fusion F protein is immunogenic and protective against challenge in cotton rats. J. Virol..

[94] Wright P.F., Ikizler M.R., Gonzales R.A., Carroll K.N., Johnson J.E., Werkhaven J.A. (2005). Growth of respiratory syncytial virus in primary epithelial cells from the human respiratory tract. J. Virol..

[95] Chung J.R., Flannery B., Thompson M.G., Gaglani M., Jackson M.L., Monto A.S., Nowalk M.P., Talbot H.K., Treanor J.J., Belongia E.A. (2016). Seasonal effectiveness of live attenuated and inactivated influenza vaccine. Pediatrics.

[96] Gaglani M., Pruszynski J., Murthy K., Clipper L., Robertson A., Reis M., Chung J.R., Piedra P.A., Avadhanula V., Nowalk M.P. (2016). Influenza vaccine effectiveness against 2009 pandemic influenza A(H1N1) virus differed by vaccine type during 2013–2014 in the United States. J. Infect. Dis..

[97] Grohskopf L.A., Sokolow L.Z., Olsen S.J., Bresee J.S., Broder K.R., Karron R.A. (2015). Prevention and control of influenza with vaccines: Recommendations of the advisory committee on immunization practices, United States, 2015–2016 influenza season. MMWR Morb. Mortal. Wkly. Rep..

[98] Grohskopf L.A., Sokolow L.Z., Broder K.R., Olsen S.J., Karron R.A., Jernigan D.B., Bresee J.S. (2016). Prevention and control of seasonal influenza with vaccines. MMWR Recomm. Rep..

[99] Grohskopf L.A., Sokolow L.Z., Broder K.R., Walter E.B., Bresee J.S., Fry A.M., Jernigan D.B. (2017). Prevention and control of seasonal influenza with vaccines: Recommendations of the advisory committee on immunization practices—United States, 2017–2018 influenza season. MMWR Recomm. Rep..

[100] Boda B., Benaoudia S., Huang S., Bonfante R., Wiszniewski L., Tseligka E.D., Tapparel C., Constant S. (2018). Antiviral drug screening by assessing epithelial functions and innate immune responses in human 3D airway epithelium model. Antivir. Res..

[101] Toots M., Yoon J.J., Cox R.M., Hart M., Sticher Z.M., Makhsous N., Plesker R., Barrena A.H., Reddy P.G., Mitchell D.G. (2019). Characterization of orally efficacious influenza drug with high resistance barrier in ferrets and human airway epithelia. Sci. Transl. Med..

[102] Sheahan T.P., Sims A.C., Zhou S., Graham R.L., Pruijssers A.J., Agostini M.L., Leist S.R., Schäfer A., Dinnon K.H., Stevens L.J. (2020). An orally bioavailable broad-spectrum antiviral inhibits SARS-CoV-2 in human airway epithelial cell cultures and multiple coronaviruses in mice. Sci. Transl. Med..

[103] DeVincenzo J., Tait D., Efthimiou J., Mori J., Kim Y.I., Thomas E., Wilson L., Harland R., Mathews N., Cockerill S. (2020). A randomized, placebo-controlled, respiratory syncytial virus human challenge study of the antiviral efficacy, safety, and pharmacokinetics of RV521, an inhibitor of the RSV-F protein. Antimicrob. Agents Chemother..

[104] Memoli M.J., Czajkowski L., Reed S., Athota R., Bristol T., Proudfoot K., Fargis S., Stein M., Dunfee R.L., Shaw P.A. (2015). Validation of the wild-type influenza A human challenge model H1N1pdMIST: An A(H1N1)pdm09 dose-finding investigational new drug study. Clin. Infect. Dis..

[105] Peretz J., Pekosz A., Lane A.P., Klein S.L. (2016). Estrogenic compounds reduce influenza A virus replication in primary human nasal epithelial cells derived from female, but not male, donors. Am. J. Physiol. Lung Cell. Mol. Physiol..

[106] Casimir G.J., Lefèvre N., Corazza F., Duchateau J. (2013). Sex and inflammation in respiratory diseases: A clinical viewpoint. Biol. Sex. Differ..

[107] Huang C.G., Lee L.A., Wu Y.C., Hsiao M.J., Horng J.T., Kuo R.L., Huang C.H., Lin Y.C., Tsao K.C., Chen M.C. (2018). A pilot study on primary cultures of human respiratory tract epithelial cells to predict patients’ responses to H7N9 infection. Oncotarget.

[108] Honce R., Karlsson E.A., Wohlgemuth N., Estrada L.D., Meliopoulos V.A., Yao J., Schultz-Cherry S. (2020). Obesity-related microenvironment promotes emergence of virulent influenza virus strains. MBio.

[109] Zhang L., Bukreyev A., Thompson C.I., Watson B., Peeples M.E., Collins P.L., Pickles R.J. (2005). Infection of ciliated cells by human parainfluenza virus type 3 in an in vitro model of human airway epithelium. J. Virol..

[110] Milewska A., Kula-Pacurar A., Wadas J., Suder A., Szczepanski A., Dabrowska A., Owczarek K., Marcello A., Ochman M., Stacel T. (2020). Replication of Severe Acute Respiratory Syndrome Coronavirus 2 in Human Respiratory Epithelium. J. Virol..

[111] Griggs T.F., Bochkov Y.A., Basnet S., Pasic T.R., Brockman-Schneider R.A., Palmenberg A.C., Gern J.E. (2017). Rhinovirus C targets ciliated airway epithelial cells. Respir. Res..

[112] Matrosovich M.N., Matrosovich T.Y., Gray T., Roberts N.A., Klenk H.-D. (2004). Human and avian influenza viruses target different cell types in cultures of human airway epithelium. Proc. Natl. Acad. Sci. USA.

[113] Warner S.M., Wiehler S., Michi A.N., Proud D. (2019). Rhinovirus replication and innate immunity in highly differentiated human airway epithelial cells. Respir. Res..

[114] Ehre C., Worthington E.N., Liesman R.M., Grubb B.R., Barbier D., O’Neal W.K., Sallenave J.M., Pickles R.J., Boucher R.C. (2012). Overexpressing mouse model demonstrates the protective role of Muc5ac in the lungs. Proc. Natl. Acad. Sci. USA.

[115] McAuley J.L., Corcilius L., Tan H.X., Payne R.J., McGuckin M.A., Brown L.E. (2017). The cell surface mucin MUC1 limits the severity of influenza A virus infection. Mucosal Immunol..

[116] Holly M.K., Diaz K., Smith J.G. (2017). Defensins in viral infection and pathogenesis. Annu. Rev. Virol..

[117] Doss M., White M.R., Tecle T., Gantz D., Crouch E.C., Jung G., Ruchala P., Waring A.J., Lehrer R.I., Hartshorn K.L. (2009). Interactions of alpha-, beta-, and theta-defensins with influenza A virus and surfactant protein D. J. Immunol..

[118] Kota S., Sabbah A., Chang T.H., Harnack R., Xiang Y., Meng X., Bose S. (2008). Role of human beta-defensin-2 during tumor necrosis factor-alpha/NF-kappaB-mediated innate antiviral response against human respiratory syncytial virus. J. Biol. Chem..

[119] Zhao H., Zhou J., Zhang K., Chu H., Liu D., Poon V.K.M., Chan C.C.S., Leung H.C., Fai N., Lin Y.P. (2016). A novel peptide with potent and broad-spectrum antiviral activities against multiple respiratory viruses. Sci. Rep..

[120] Bertram S., Glowacka I., Müller M.A., Lavender H., Gnirss K., Nehlmeier I., Niemeyer D., He Y., Simmons G., Drosten C. (2011). Cleavage and activation of the severe acute respiratory syndrome coronavirus spike protein by human airway trypsin-like protease. J. Virol..

[121] Hoffmann M., Kleine-Weber H., Schroeder S., Krüger N., Herrler T., Erichsen S., Schiergens T.S., Herrler G., Wu N.H., Nitsche A. (2020). SARS-CoV-2 cell entry depends on ACE2 and TMPRSS2 and is blocked by a clinically proven protease inhibitor. Cell.

[122] Xia S., Lan Q., Su S., Wang X., Xu W., Liu Z., Zhu Y., Wang Q., Lu L., Jiang S. (2020). The role of furin cleavage site in SARS-CoV-2 spike protein-mediated membrane fusion in the presence or absence of trypsin. Signal Transduct. Target. Ther..

[123] Meyer M., Jaspers I. (2015). Respiratory protease/antiprotease balance determines susceptibility to viral infection and can be modified by nutritional antioxidants. Am. J. Physiol. Lung Cell. Mol. Physiol..

[124] Sakai K., Ami Y., Tahara M., Kubota T., Anraku M., Abe M., Nakajima N., Sekizuka T., Shirato K., Suzaki Y. (2014). The host protease TMPRSS2 plays a major role in in vivo replication of emerging H7N9 and seasonal influenza viruses. J. Virol..

[125] Hatesuer B., Bertram S., Mehnert N., Bahgat M.M., Nelson P.S., Pöhlmann S., Schughart K. (2013). Tmprss2 is essential for influenza H1N1 virus pathogenesis in mice. PLoS Pathog..

[126] Kühn N., Bergmann S., Kösterke N., Lambertz R.L.O., Keppner A., van den Brand J.M.A., Pöhlmann S., Weiß S., Hummler E., Hatesuer B. (2016). The proteolytic activation of (H3N2) influenza A virus hemagglutinin is facilitated by different type II transmembrane serine proteases. J. Virol..

[127] Millet J.K., Whittaker G.R. (2015). Host cell proteases: Critical determinants of coronavirus tropism and pathogenesis. Virus Res..

[128] Zhou Y., Vedantham P., Lu K., Agudelo J., Carrion R., Nunneley J.W., Barnard D., Pöhlmann S., McKerrow J.H., Renslo A.R. (2015). Protease inhibitors targeting coronavirus and filovirus entry. Antivir. Res..

[129] Zhou N., Pan T., Zhang J., Li Q., Zhang X., Bai C., Huang F., Peng T., Zhang J., Liu C. (2016). Glycopeptide antibiotics potently inhibit cathepsin L in the late endosome/lysosome and block the entry of Ebola virus, Middle East respiratory syndrome coronavirus (MERS-CoV), and severe acute respiratory syndrome coronavirus (SARS-CoV). J. Biol. Chem..

[130] Shirato K., Kawase M., Matsuyama S. (2018). Wild-type human coronaviruses prefer cell-surface TMPRSS2 to endosomal cathepsins for cell entry. Virology.

[131] Xu X., Greenland J.R., Gotts J.E., Matthay M.A., Caughey G.H. (2016). Cathepsin L helps to defend mice from infection with influenza, A. PLoS ONE.

[132] Coleman M.D., Ha S.D., Haeryfar S.M.M., Barr S.D., Kim S.O. (2018). Cathepsin B plays a key role in optimal production of the influenza A virus. J. Virol. Antivir. Res..

[133] Garten W., Braden C., Arendt A., Peitsch C., Baron J., Lu Y., Pawletko K., Hardes K., Steinmetzer T., Böttcher-Friebertshäuser E. (2015). Influenza virus activating host proteases: Identification, localization and inhibitors as potential therapeutics. Eur. J. Cell Biol..

[134] Tarnow C., Engels G., Arendt A., Schwalm F., Sediri H., Preuss A., Nelson P.S., Garten W., Klenk H.D., Gabriel G. (2014). TMPRSS2 is a host factor that is essential for pneumotropism and pathogenicity of H7N9 influenza A virus in mice. J. Virol..

[135] Beaulieu A., Gravel É., Cloutier A., Marois I., Colombo É., Désilets A., Verreault C., Leduc R., Marsault É., Richter M.V. (2013). Matriptase proteolytically activates influenza virus and promotes multicycle replication in the human airway epithelium. J. Virol..

[136] Lambertz R.L.O., Gerhauser I., Nehlmeier I., Leist S.R., Kollmus H., Pöhlmann S., Schughart K. (2019). Tmprss2 knock-out mice are resistant to H10 influenza A virus pathogenesis. J. Gen. Virol..

[137] Rojas-Quintero J., Wang X., Tipper J., Burkett P.R., Zuñiga J., Ashtekar A.R., Polverino F., Rout A., Yambayev I., Hernández C. (2018). Matrix metalloproteinase-9 deficiency protects mice from severe influenza A viral infection. JCI Insight.

[138] Dabo A.J., Cummins N., Eden E., Geraghty P. (2015). Matrix Metalloproteinase 9 exerts antiviral activity against respiratory syncytial virus. PLoS ONE.

[139] Kong M.Y.F., Whitley R.J., Peng N., Oster R., Schoeb T.R., Sullender W., Ambalavanan N., Clancy J.P., Gaggar A., Blalock J.E. (2015). Matrix Metalloproteinase-9 mediates RSV infection in vitro and in vivo. Viruses.

[140] Dittmann M., Hoffmann H.H., Scull M.A., Gilmore R.H., Bell K.L., Ciancanelli M., Wilson S.J., Crotta S., Yu Y., Flatley B. (2015). A serpin shapes the extracellular environment to prevent influenza A virus maturation. Cell..

[141] Wakabayashi H., Oda H., Yamauchi K., Abe F. (2014). Lactoferrin for prevention of common viral infections. J. Infect. Chemother..

[142] Kesimer M., Scull M., Brighton B., DeMaria G., Burns K., O’Neal W., Pickles R.J., Sheehan J.K. (2009). Characterization of exosome-like vesicles released from human tracheobronchial ciliated epithelium: A possible role in innate defense. FASEB J..

[143] Ichinohe T., Pang I.K., Kumamoto Y., Peaper D.R., Ho J.H., Murray T.S., Iwasaki A. (2011). Microbiota regulates immune defense against respiratory tract influenza A virus infection. Proc. Natl. Acad. Sci. USA.

[144] Button B., Cai L.H., Ehre C., Kesimer M., Hill D.B., Sheehan J.K., Boucher R.C., Rubinstein M. (2012). A periciliary brush promotes the lung health by separating the mucus layer from airway epithelia. Science.

[145] Song Y., Namkung W., Nielson D.W., Lee J.-W., Finkbeiner W.E., Verkman A.S. (2009). Airway surface liquid depth measured in ex vivo fragments of pig and human trachea: Dependence on Na+ and Cl- channel function. Am. J. Physiol. Lung Cell. Mol. Physiol..

[146] Jayaraman S., Song Y., Vetrivel L., Shankar L., Verkman A.S. (2001). Noninvasive in vivo fluorescence measurement of airway-surface liquid depth, salt concentration, and pH. J. Clin. Investig..

[147] Boucher R.C. (2007). Airway surface dehydration in cystic fibrosis: Pathogenesis and therapy. Annu. Rev. Med..

[148] Chen E.Y.T., Yang N., Quinton P.M., Chin W.-C. (2010). A new role for bicarbonate in mucus formation. Am. J. Physiol. Lung Cell. Mol. Physiol..

[149] Tang X.X., Ostedgaard L.S., Hoegger M.J., Moninger T.O., Karp P.H., McMenimen J.D., Choudhury B., Varki A., Stoltz D.A., Welsh M.J. (2016). Acidic pH increases airway surface liquid viscosity in cystic fibrosis. J. Clin. Investig..

[150] Fischer H., Widdicombe J.H. (2006). Mechanisms of acid and base secretion by the airway epithelium. J. Membr. Biol..

[151] Ermund A., Meiss L.N., Rodriguez-Pineiro A.M., Bähr A., Nilsson H.E., Trillo-Muyo S., Ridley C., Thornton D.J., Wine J.J., Hebert H. (2017). The normal trachea is cleaned by MUC5B mucin bundles from the submucosal glands coated with the MUC5AC mucin. Biochem. Biophys. Res. Commun..

[152] Widdicombe J.H., Wine J.J. (2015). Airway gland structure and function. Physiol. Rev..

[153] Okuda K., Chen G., Subramani D.B., Wolf M., Gilmore R.C., Kato T., Radicioni G., Kesimer M., Chua M., Dang H. (2019). Localization of secretory mucins MUC5AC and MUC5B in normal/healthy human airways. Am. J. Respir. Crit. Care Med..

[154] Amini S.E., Gouyer V., Portal C., Gottrand F., Desseyn J.L. (2019). Muc5b is mainly expressed and sialylated in the nasal olfactory epithelium whereas Muc5ac is exclusively expressed and fucosylated in the nasal respiratory epithelium. Histochem. Cell Biol..

[155] Roy M.G., Livraghi-Butrico A., Fletcher A.A., McElwee M.M., Evans S.E., Boerner R.M., Alexander S.N., Bellinghausen L.K., Song A.S., Petrova Y.M. (2014). Muc5b is required for airway defence. Nature.

[156] Hancock L.A., Hennessy C.E., Solomon G.M., Dobrinskikh E., Estrella A., Hara N., Hill D.B., Kissner W.J., Markovetz M.R., Grove Villalon D.E. (2018). Muc5b overexpression causes mucociliary dysfunction and enhances lung fibrosis in mice. Nat. Commun..

[157] Zhang Q., Wang Y., Qu D., Yu J., Yang J. (2019). The possible pathogenesis of idiopathic pulmonary fibrosis considering MUC5B. Biomed. Res. Int..

[158] Ridley C., Thornton D.J. (2018). Mucins: The frontline defence of the lung. Biochem. Soc. Trans..

[159] Evans C.M., Raclawska D.S., Ttofali F., Liptzin D.R., Fletcher A.A., Harper D.N., McGing M.A., McElwee M.M., Williams O.W., Sanchez E. (2015). The polymeric mucin Muc5ac is required for allergic airway hyperreactivity. Nat. Commun..

[160] Lachowicz-Scroggins M.E., Yuan S., Kerr S.C., Dunican E.M., Yu M., Carrington S.D., Fahy J.V. (2016). Abnormalities in MUC5AC and MUC5B protein in airway mucus in asthma. Am. J. Respir. Crit. Care Med..

[161] Bonser L.R., Erle D.J. (2017). Airway mucus and asthma: The role of MUC5AC and MUC5B. J. Clin. Med. Res..

[162] Peñia M.T., Aujla P.K., Zudaire E., Watson A.M., Przygodzki R., Zalzal G.H., Rose M.C. (2007). Localization and expression of MUC5B and MUC7 mucins in pediatric sinus mucosa. Ann. Otol. Rhinol. Laryngol..

[163] Martínez-Antón A., Debolós C., Garrido M., Roca-Ferrer J., Barranco C., Alobid I., Xaubet A., Picado C., Mullol J. (2006). Mucin genes have different expression patterns in healthy and diseased upper airway mucosa. Clin. Exp. Allergy.

[164] Cha H.J., Song K.S. (2018). Effect of MUC8 on airway inflammation: A friend or a foe?. J. Clin. Med. Res..

[165] Cha H.J., Jung M.S., Ahn D.W., Choi J.K., Ock M.S., Kim K.S., Yoon J.H., Song E.J., Song K.S. (2015). Silencing of MUC8 by siRNA increases P2Y₂-induced airway inflammation. Am. J. Physiol. Lung Cell. Mol. Physiol..

[166] van Putten J.P.M., Strijbis K. (2017). Transmembrane mucins: Signaling receptors at the intersection of inflammation and cancer. J. Innate Immun..

[167] Gipson I.K., Spurr-Michaud S., Tisdale A., Menon B.B. (2014). Comparison of the transmembrane mucins MUC1 and MUC16 in epithelial barrier function. PLoS ONE.

[168] Ueno K., Koga T., Kato K., Golenbock D.T., Gendler S.J., Kai H., Kim K.C. (2008). MUC1 mucin is a negative regulator of toll-like receptor signaling. Am. J. Respir. Cell Mol. Biol..

[169] Kato K., Lillehoj E.P., Lu W., Kim K.C. (2017). MUC1: The first respiratory mucin with an anti-inflammatory function. J. Clin. Med. Res..

[170] Mahanta S., Fessler S.P., Park J., Bamdad C. (2008). A minimal fragment of MUC1 mediates growth of cancer cells. PLoS ONE.

[171] Higuchi T., Orita T., Nakanishi S., Katsuya K., Watanabe H., Yamasaki Y., Waga I., Nanayama T., Yamamoto Y., Munger W. (2004). Molecular cloning, genomic structure, and expression analysis of MUC20, a novel mucin protein, up-regulated in injured kidney. J. Biol. Chem..

[172] Walters R.W., Pilewski J.M., Chiorini J.A., Zabner J. (2002). Secreted and transmembrane mucins inhibit gene transfer with AAV4 more efficiently than AAV5. J. Biol. Chem..

[173] Corfield A.P. (2015). Mucins: A biologically relevant glycan barrier in mucosal protection. Biochim. Biophys. Acta.

[174] Priyadharshini V.S., Ramírez-Jiménez F., Molina-Macip M., Renteria-Rosales C., Santiago-Cruz J., Zarate-Segura P., Lara-Padilla E., Teran L.M. (2018). Human Neutrophil Defensin-1, -3, and -4 are elevated in nasal aspirates from children with naturally occurring adenovirus infection. Can. Respir. J..

[175] Rohde G., Message S.D., Haas J.J., Kebadze T., Parker H., Laza-Stanca V., Khaitov M.R., Kon O.M., Stanciu L.A., Mallia P. (2014). CXC chemokines and antimicrobial peptides in rhinovirus-induced experimental asthma exacerbations. Clin. Exp. Allergy.

[176] Gu J., Huang Y. (2017). β-Defensin-2 is overexpressed in human vocal cord polyps. Eur. Arch. Otorhinolaryngol..

[177] Taylor K., Clarke D.J., McCullough B., Chin W., Seo E., Yang D., Oppenheim J., Uhrin D., Govan J.R.W., Campopiano D.J. (2008). Analysis and separation of residues important for the chemoattractant and antimicrobial activities of beta-defensin 3. J. Biol. Chem..

[178] Funderburg N.T., Jadlowsky J.K., Lederman M.M., Feng Z., Weinberg A., Sieg S.F. (2011). The Toll-like receptor 1/2 agonists Pam(3) CSK(4) and human β-defensin-3 differentially induce interleukin-10 and nuclear factor-κB signalling patterns in human monocytes. Immunology.

[179] Nagaoka I., Niyonsaba F., Tsutsumi-Ishii Y., Tamura H., Hirata M. (2008). Evaluation of the effect of human beta-defensins on neutrophil apoptosis. Int. Immunol..

[180] Semple F., Webb S., Li H.-N., Patel H.B., Perretti M., Jackson I.J., Gray M., Davidson D.J., Dorin J.R. (2010). Human beta-defensin 3 has immunosuppressive activity in vitro and in vivo. Eur. J. Immunol..

[181] Shen Z., Zhou Y., Qu L., Lei H. (2017). ATP serves an anti-inflammatory role by enhancing β-defensin-2 response in acute pneumonia of rat. Biomed. Rep..

[182] Cui D., Lyu J., Li H., Lei L., Bian T., Li L., Yan F. (2017). Human β-defensin 3 inhibits periodontitis development by suppressing inflammatory responses in macrophages. Mol. Immunol..

[183] Semple F., Dorin J.R. (2012). β-Defensins: Multifunctional modulators of infection, inflammation and more?. J. Innate Immun..

[184] Lehrer R.I., Cole A.M., Selsted M.E. (2012). θ-Defensins: Cyclic peptides with endless potential. J. Biol. Chem..

[185] Lu P., Takai K., Weaver V.M., Werb Z. (2011). Extracellular matrix degradation and remodeling in development and disease. Cold Spring Harb. Perspect. Biol..

[186] Yue J., Zhang K., Chen J. (2012). Role of integrins in regulating proteases to mediate extracellular matrix remodeling. Cancer Microenviron..

[187] Knaapi J., Kiviranta R., Laine J., Kääpä P., Lukkarinen H. (2015). Cathepsin K overexpression modifies lung development in newborn mice. Pediatr. Pulmonol..

[188] Abboud R.T., Vimalanathan S. (2008). Pathogenesis of COPD. Part, I. The role of protease-antiprotease imbalance in emphysema. Int. J. Tuberc. Lung Dis..

[189] Ashley S.L., Xia M., Murray S., O’Dwyer D.N., Grant E., White E.S., Flaherty K.R., Martinez F.J., Moore B.B. (2016). Six-SOMAmer index relating to immune, protease and angiogenic functions predicts progression in IPF. PLoS ONE.

[190] Nikaido T., Tanino Y., Wang X., Sato Y., Togawa R., Kikuchi M., Misa K., Saito K., Fukuhara N., Kawamata T. (2018). Serum decorin is a potential prognostic biomarker in patients with acute exacerbation of idiopathic pulmonary fibrosis. J. Thorac. Dis..

[191] Kehlet S.N., Bager C.L., Willumsen N., Dasgupta B., Brodmerkel C., Curran M., Brix S., Leeming D.J., Karsdal M.A. (2017). Cathepsin-S degraded decorin are elevated in fibrotic lung disorders—Development and biological validation of a new serum biomarker. BMC Pulm. Med..

[192] Yuan L., Zou C., Ge W., Liu Y., Hu B., Wang J., Lin B., Li Y., Ma E. (2020). A novel cathepsin L inhibitor prevents the progression of idiopathic pulmonary fibrosis. Bioorg. Chem..

[193] Weldon S., McNally P., McAuley D.F., Oglesby I.K., Wohlford-Lenane C.L., Bartlett J.A., Scott C.J., McElvaney N.G., Greene C.M., McCray P.B. (2014). miR-31 dysregulation in cystic fibrosis airways contributes to increased pulmonary cathepsin S production. Am. J. Respir. Crit. Care Med..

[194] Small D.M., Brown R.R., Doherty D.F., Abladey A., Zhou-Suckow Z., Delaney R.J., Kerrigan L., Dougan C.M., Borensztajn K.S., Holsinger L. (2019). Targeting of cathepsin S reduces cystic fibrosis-like lung disease. Eur. Respir. J..

[195] Laguna T.A., Williams C.B., Nunez M.G., Welchlin-Bradford C., Moen C.E., Reilly C.S., Wendt C.H. (2018). Biomarkers of inflammation in infants with cystic fibrosis. Respir. Res..

[196] Nakajima T., Nakamura H., Owen C.A., Yoshida S., Tsuduki K., Chubachi S., Shirahata T., Mashimo S., Nakamura M., Takahashi S. (2016). Plasma cathepsin S and cathepsin S/cystatin C ratios are potential biomarkers for COPD. Dis. Markers.

[197] Zhou P.P., Zhang W.Y., Li Z.F., Chen Y.R., Kang X.C., Jiang Y.X. (2016). Association between SNPs in the promoter region in cathepsin S and risk of asthma in Chinese Han population. Eur. Rev. Med. Pharmacol. Sci..

[198] Craig V.J., Zhang L., Hagood J.S., Owen C.A. (2015). Matrix metalloproteinases as therapeutic targets for idiopathic pulmonary fibrosis. Am. J. Respir. Cell Mol. Biol..

[199] Yamashita C.M., Radisky D.C., Aschner Y., Downey G.P. (2014). The importance of matrix metalloproteinase-3 in respiratory disorders. Expert Rev. Respir. Med..

[200] McKeown S., Richter A.G., O’Kane C., McAuley D.F., Thickett D.R. (2009). MMP expression and abnormal lung permeability are important determinants of outcome in IPF. Eur. Respir. J..

[201] Navratilova Z., Kolek V., Petrek M. (2016). Matrix Metalloproteinases and their inhibitors in chronic obstructive pulmonary disease. Arch. Immunol. Ther. Exp..

[202] Grzela K., Litwiniuk M., Zagorska W., Grzela T. (2016). Airway remodeling in chronic obstructive pulmonary disease and asthma: The role of Matrix Metalloproteinase-9. Arch. Immunol. Ther. Exp..

[203] Chokki M., Yamamura S., Eguchi H., Masegi T., Horiuchi H., Tanabe H., Kamimura T., Yasuoka S. (2004). Human airway trypsin-like protease increases mucin gene expression in airway epithelial cells. Am. J. Respir. Cell Mol. Biol..

[204] Zuo K., Qi Y., Yuan C., Jiang L., Xu P., Hu J., Huang M., Li J. (2019). Specifically targeting cancer proliferation and metastasis processes: The development of matriptase inhibitors. Cancer Metastasis Rev..

[205] Shi G.P., Villadangos J.A., Dranoff G., Small C., Gu L., Haley K.J., Riese R., Ploegh H.L., Chapman H.A. (1999). Cathepsin S required for normal MHC class II peptide loading and germinal center development. Immunity.

[206] Driessen C., Bryant R.A., Lennon-Duménil A.M., Villadangos J.A., Bryant P.W., Shi G.P., Chapman H.A., Ploegh H.L. (1999). Cathepsin S controls the trafficking and maturation of MHC class II molecules in dendritic cells. J. Cell Biol..

[207] Nakagawa T., Roth W., Wong P., Nelson A., Farr A., Deussing J., Villadangos J.A., Ploegh H., Peters C., Rudensky A.Y. (1998). Cathepsin L: Critical role in Ii degradation and CD4 T cell selection in the thymus. Science.

[208] Zhang N., Gao P., Yin B., Li J., Wu T., Kuang Y., Wu W., Li J. (2019). Cathepsin L promotes secretory IgA response by participating in antigen presentation pathways during Mycoplasma Hyopneumoniae infection. PLoS ONE.

[209] Sevenich L., Hagemann S., Stoeckle C., Tolosa E., Peters C., Reinheckel T. (2010). Expression of human cathepsin L or human cathepsin V in mouse thymus mediates positive selection of T helper cells in cathepsin L knock-out mice. Biochimie.

[210] Matsumoto F., Saitoh S.I., Fukui R., Kobayashi T., Tanimura N., Konno K., Kusumoto Y., Akashi-Takamura S., Miyake K. (2008). Cathepsins are required for Toll-like receptor 9 responses. Biochem. Biophys. Res. Commun..

[211] Patel S., Homaei A., El-Seedi H.R., Akhtar N. (2018). Cathepsins: Proteases that are vital for survival but can also be fatal. Biomed. Pharmacother..

[212] Costa M.G.S., Batista P.R., Shida C.S., Robert C.H., Bisch P.M., Pascutti P.G. (2010). How does heparin prevent the pH inactivation of cathepsin B? Allosteric mechanism elucidated by docking and molecular dynamics. BMC Genom..

[213] Greenlee K.J., Werb Z., Kheradmand F. (2007). Matrix metalloproteinases in lung: Multiple, multifarious, and multifaceted. Physiol. Rev..

[214] Masumoto K., de Rooij J.D., Suita S., Rottier R., Tibboel D., de Krijger R.R. (2005). Expression of matrix metalloproteinases and tissue inhibitors of metalloproteinases during normal human pulmonary development. Histopathology.

[215] Hendrix A.Y., Kheradmand F. (2017). The role of Matrix Metalloproteinases in development, repair, and destruction of the lungs. Prog. Mol. Biol. Transl. Sci..

[216] Brilha S., Chong D.L.W., Khawaja A.A., Ong C.W.M., Guppy N.J., Porter J.C., Friedland J.S. (2018). Integrin α2β1 expression regulates Matrix Metalloproteinase-1-dependent bronchial epithelial repair in pulmonary tuberculosis. Front. Immunol..

[217] Herrera I., Cisneros J., Maldonado M., Ramírez R., Ortiz-Quintero B., Anso E., Chandel N.S., Selman M., Pardo A. (2013). Matrix metalloproteinase (MMP)-1 induces lung alveolar epithelial cell migration and proliferation, protects from apoptosis, and represses mitochondrial oxygen consumption. J. Biol. Chem..

[218] Blázquez-Prieto J., López-Alonso I., Amado-Rodríguez L., Huidobro C., González-López A., Kuebler W.M., Albaiceta G.M. (2018). Impaired lung repair during neutropenia can be reverted by matrix metalloproteinase-9. Thorax.

[219] Howell C., Smith J.R., Shute J.K. (2018). Targeting matrix metalloproteinase-13 in bronchial epithelial repair. Clin. Exp. Allergy.

[220] Rohani M.G., Parks W.C. (2015). Matrix remodeling by MMPs during wound repair. Matrix Biol..

[221] Fujita Y., Kadota T., Araya J., Ochiya T., Kuwano K. (2018). Extracellular vesicles: New players in lung immunity. Am. J. Respir. Cell Mol. Biol..

[222] Mueller S.K., Nocera A.L., Bleier B.S. (2018). Exosome function in aerodigestive mucosa. Nanomedicine.

[223] Valadi H., Ekström K., Bossios A., Sjöstrand M., Lee J.J., Lötvall J.O. (2007). Exosome-mediated transfer of mRNAs and microRNAs is a novel mechanism of genetic exchange between cells. Nat. Cell Biol..

[224] Schageman J., Zeringer E., Li M., Barta T., Lea K., Gu J., Magdaleno S., Setterquist R., Vlassov A.V. (2013). The complete exosome workflow solution: From isolation to characterization of RNA cargo. Biomed. Res. Int..

[225] Kulshreshtha A., Ahmad T., Agrawal A., Ghosh B. (2013). Proinflammatory role of epithelial cell-derived exosomes in allergic airway inflammation. J. Allergy Clin. Immunol..

[226] Bourdonnay E., Zasłona Z., Penke L.R.K., Speth J.M., Schneider D.J., Przybranowski S., Swanson J.A., Mancuso P., Freeman C.M., Curtis J.L. (2015). Transcellular delivery of vesicular SOCS proteins from macrophages to epithelial cells blunts inflammatory signaling. J. Exp. Med..

[227] Torregrosa Paredes P., Esser J., Admyre C., Nord M., Rahman Q.K., Lukic A., Rådmark O., Grönneberg R., Grunewald J., Eklund A. (2012). Bronchoalveolar lavage fluid exosomes contribute to cytokine and leukotriene production in allergic asthma. Allergy.

[228] Nocera A.L., Miyake M.M., Seifert P., Han X., Bleier B.S. (2017). Exosomes mediate interepithelial transfer of functional P-glycoprotein in chronic rhinosinusitis with nasal polyps. Laryngoscope.

[229] Wahlund C.J.E., Eklund A., Grunewald J., Gabrielsson S. (2017). Pulmonary extracellular vesicles as mediators of local and systemic inflammation. Front. Cell Dev. Biol..

[230] Wang Y.Y., Harit D., Subramani D.B., Arora H., Kumar P.A., Lai S.K. (2017). Influenza-binding antibodies immobilise influenza viruses in fresh human airway mucus. Eur. Respir. J..

[231] Kobayashi K., Suzukawa M., Watanabe K., Arakawa S., Igarashi S., Asari I., Hebisawa A., Matsui H., Nagai H., Nagase T. (2020). Secretory IgA accumulated in the airspaces of idiopathic pulmonary fibrosis and promoted VEGF, TGF-β and IL-8 production by A549 cells. Clin. Exp. Immunol..

[232] Cereser L., De Carli M., d’Angelo P., Zanelli E., Zuiani C., Girometti R. (2018). High-resolution computed tomography findings in humoral primary immunodeficiencies and correlation with pulmonary function tests. World J. Radiol..

[233] Charlson E.S., Bittinger K., Haas A.R., Fitzgerald A.S., Frank I., Yadav A., Bushman F.D., Collman R.G. (2011). Topographical continuity of bacterial populations in the healthy human respiratory tract. Am. J. Respir. Crit. Care Med..

[234] de Steenhuijsen Piters W.A.A., Sanders E.A.M., Bogaert D. (2015). The role of the local microbial ecosystem in respiratory health and disease. Philos. Trans. R. Soc. Lond. B Biol. Sci..

[235] Ling Z., Liu X., Luo Y., Yuan L., Nelson K.E., Wang Y., Xiang C., Li L. (2013). Pyrosequencing analysis of the human microbiota of healthy Chinese undergraduates. BMC Genom..

[236] Charlson E.S., Chen J., Custers-Allen R., Bittinger K., Li H., Sinha R., Hwang J., Bushman F.D., Collman R.G. (2010). Disordered microbial communities in the upper respiratory tract of cigarette smokers. PLoS ONE.

[237] Yi H., Yong D., Lee K., Cho Y.J., Chun J. (2014). Profiling bacterial community in upper respiratory tracts. BMC Infect. Dis..

[238] Charlson E.S., Bittinger K., Chen J., Diamond J.M., Li H., Collman R.G., Bushman F.D. (2012). Assessing bacterial populations in the lung by replicate analysis of samples from the upper and lower respiratory tracts. PLoS ONE.

[239] Dickson R.P., Erb-Downward J.R., Freeman C.M., Walker N., Scales B.S., Beck J.M., Martinez F.J., Curtis J.L., Lama V.N., Huffnagle G.B. (2014). Changes in the lung microbiome following lung transplantation include the emergence of two distinct Pseudomonas species with distinct clinical associations. PLoS ONE.

[240] Venkataraman A., Bassis C.M., Beck J.M., Young V.B., Curtis J.L., Huffnagle G.B., Schmidt T.M. (2015). Application of a neutral community model to assess structuring of the human lung microbiome. MBio.

[241] Bassis C.M., Erb-Downward J.R., Dickson R.P., Freeman C.M., Schmidt T.M., Young V.B., Beck J.M., Curtis J.L., Huffnagle G.B. (2015). Analysis of the upper respiratory tract microbiotas as the source of the lung and gastric microbiotas in healthy individuals. MBio.

[242] Marsh R.L., Kaestli M., Chang A.B., Binks M.J., Pope C.E., Hoffman L.R., Smith-Vaughan H.C. (2016). The microbiota in bronchoalveolar lavage from young children with chronic lung disease includes taxa present in both the oropharynx and nasopharynx. Microbiome.

[243] de Steenhuijsen Piters W.A.A., Heinonen S., Hasrat R., Bunsow E., Smith B., Suarez-Arrabal M.-C., Chaussabel D., Cohen D.M., Sanders E.A.M., Ramilo O. (2016). Nasopharyngeal Microbiota, host transcriptome, and disease severity in children with respiratory syncytial virus infection. Am. J. Respir. Crit. Care Med..

[244] Korten I., Mika M., Klenja S., Kieninger E., Mack I., Barbani M.T., Gorgievski M., Frey U., Hilty M., Latzin P. (2016). Interactions of respiratory viruses and the nasal microbiota during the first year of life in healthy infants. mSphere.

[245] Toivonen L., Camargo C.A., Gern J.E., Bochkov Y.A., Mansbach J.M., Piedra P.A., Hasegawa K. (2019). Association between rhinovirus species and nasopharyngeal microbiota in infants with severe bronchiolitis. J. Allergy Clin. Immunol..

[246] Wen Z., Xie G., Zhou Q., Qiu C., Li J., Hu Q., Dai W., Li D., Zheng Y., Wen F. (2018). Distinct nasopharyngeal and oropharyngeal microbiota of children with influenza A virus compared with healthy children. Biomed. Res. Int..

[247] Edouard S., Million M., Bachar D., Dubourg G., Michelle C., Ninove L., Charrel R., Raoult D. (2018). The nasopharyngeal microbiota in patients with viral respiratory tract infections is enriched in bacterial pathogens. Eur. J. Clin. Microbiol. Infect. Dis..

[248] Poole K., Meder D., Simons K., Müller D. (2004). The effect of raft lipid depletion on microvilli formation in MDCK cells, visualized by atomic force microscopy. FEBS Lett..

[249] Wu J., Wang Y., Liu G., Jia Y., Yang J., Shi J., Dong J., Wei J., Liu X. (2017). Characterization of air-liquid interface culture of A549 alveolar epithelial cells. Braz. J. Med. Biol. Res..

[250] Joshi S., Kumar S., Bafna S., Rachagani S., Wagner K.U., Jain M., Batra S.K. (2015). Genetically engineered mucin mouse models for inflammation and cancer. Cancer Metastasis Rev..

[251] Zanin M., Baviskar P., Webster R., Webby R. (2016). The interaction between respiratory pathogens and mucus. Cell Host Microbe.

[252] Gagneux P., Cheriyan M., Hurtado-Ziola N., van der Linden E.C.M.B., Anderson D., McClure H., Varki A., Varki N.M. (2003). Human-specific regulation of alpha 2-6-linked sialic acids. J. Biol. Chem..

[253] Kim M., Mun H., Sung C.O., Cho E.J., Jeon H.-J., Chun S.-M., Jung D.J., Shin T.H., Jeong G.S., Kim D.K. (2019). Patient-derived lung cancer organoids as in vitro cancer models for therapeutic screening. Nat. Commun..

[254] Jose S.S., De Zuani M., Tidu F., Hortová Kohoutková M., Pazzagli L., Forte G., Spaccapelo R., Zelante T., Frič J. (2020). Comparison of two human organoid models of lung and intestinal inflammation reveals Toll-like receptor signalling activation and monocyte recruitment. Clin. Transl. Immunol..

[255] Stonebraker J.R., Wagner D., Lefensty R.W., Burns K., Gendler S.J., Bergelson J.M., Boucher R.C., O’Neal W.K., Pickles R.J. (2004). Glycocalyx restricts adenoviral vector access to apical receptors expressed on respiratory epithelium in vitro and in vivo: Role for tethered mucins as barriers to lumenal infection. J. Virol..

[256] Zanin M., Marathe B., Wong S.-S., Yoon S.-W., Collin E., Oshansky C., Jones J., Hause B., Webby R. (2015). Pandemic Swine H1N1 Influenza Viruses with Almost Undetectable Neuraminidase Activity Are Not Transmitted via Aerosols in Ferrets and Are Inhibited by Human Mucus but Not Swine Mucus. J. Virol..

[257] Vahey M.D., Fletcher D.A. (2019). Influenza A virus surface proteins are organized to help penetrate host mucus. eLife.

[258] Markovetz M.R., Subramani D.B., Kissner W.J., Morrison C.B., Garbarine I.C., Ghio A., Ramsey K.A., Arora H., Kumar P., Nix D.B. (2019). Endotracheal tube mucus as a source of airway mucus for rheological study. Am. J. Physiol. Lung Cell. Mol. Physiol..

[259] Schuster B.S., Suk J.S., Woodworth G.F., Hanes J. (2013). Nanoparticle diffusion in respiratory mucus from humans without lung disease. Biomaterials.

[260] Yuan S., Hollinger M., Lachowicz-Scroggins M.E., Kerr S.C., Dunican E.M., Daniel B.M., Ghosh S., Erzurum S.C., Willard B., Hazen S.L. (2015). Oxidation increases mucin polymer cross-links to stiffen airway mucus gels. Sci. Transl. Med..

[261] Innes A.L., Carrington S.D., Thornton D.J., Kirkham S., Rousseau K., Dougherty R.H., Raymond W.W., Caughey G.H., Muller S.J., Fahy J.V. (2009). Ex vivo sputum analysis reveals impairment of protease-dependent mucus degradation by plasma proteins in acute asthma. Am. J. Respir. Crit. Care Med..

[262] Hill D.B., Vasquez P.A., Mellnik J., McKinley S.A., Vose A., Mu F., Henderson A.G., Donaldson S.H., Alexis N.E., Boucher R.C. (2014). A biophysical basis for mucus solids concentration as a candidate biomarker for airways disease. PLoS ONE.

[263] Schiller J.L., Lai S.K. (2020). Tuning barrier properties of biological hydrogels. ACS Appl. Bio Mater..

[264] Frickmann H., Jungblut S., Hirche T.O., Groß U., Kuhns M., Zautner A.E. (2012). Spectrum of viral infections in patients with cystic fibrosis. Eur. J. Microbiol. Immunol..

[265] Boucher R.C. (2015). On the pathogenesis of acute exacerbations of mucoobstructive lung diseases. Ann. Am. Thorac. Soc..

[266] Duncan G.A., Jung J., Joseph A., Thaxton A.L., West N.E., Boyle M.P., Hanes J., Suk J.S. (2016). Microstructural alterations of sputum in cystic fibrosis lung disease. JCI Insight.

[267] Anderson W.H., Coakley R.D., Button B., Henderson A.G., Zeman K.L., Alexis N.E., Peden D.B., Lazarowski E.R., Davis C.W., Bailey S. (2015). The relationship of mucus concentration (hydration) to mucus osmotic pressure and transport in chronic bronchitis. Am. J. Respir. Crit. Care Med..

[268] Kesimer M., Ford A.A., Ceppe A., Radicioni G., Cao R., Davis C.W., Doerschuk C.M., Alexis N.E., Anderson W.H., Henderson A.G. (2017). Airway mucin concentration as a marker of chronic bronchitis. N. Engl. J. Med..

[269] Essaidi-Laziosi M., Brito F., Benaoudia S., Royston L., Cagno V., Fernandes-Rocha M., Piuz I., Zdobnov E., Huang S., Constant S. (2018). Propagation of respiratory viruses in human airway epithelia reveals persistent virus-specific signatures. J. Allergy Clin. Immunol..

[270] Lai S.K., Wang Y.Y., Wirtz D., Hanes J. (2009). Micro- and macrorheology of mucus. Adv. Drug Deliv. Rev..

[271] Bansil R., Turner B.S. (2018). The biology of mucus: Composition, synthesis and organization. Adv. Drug Deliv. Rev..

[272] Duncan G.A., Jung J., Hanes J., Suk J.S. (2016). The mucus barrier to inhaled gene therapy. Mol. Ther..

[273] Huck B.C., Hartwig O., Biehl A., Schwarzkopf K., Wagner C., Loretz B., Murgia X., Lehr C.-M. (2019). Macro- and microrheological properties of mucus surrogates in comparison to native intestinal and pulmonary mucus. Biomacromolecules.

[274] Xu Q., Ensign L.M., Boylan N.J., Schön A., Gong X., Yang J.C., Lamb N.W., Cai S., Yu T., Freire E. (2015). Impact of surface Polyethylene Glycol (PEG) density on biodegradable nanoparticle transport in mucus ex vivo and distribution in vivo. ACS Nano.

[275] Atanasova K.R., Reznikov L.R. (2019). Strategies for measuring airway mucus and mucins. Respir. Res..

[276] Ramsey K.A., Rushton Z.L., Ehre C. (2016). Mucin agarose gel electrophoresis: Western blotting for high-molecular-weight glycoproteins. J. Vis. Exp..

[277] Abdullah L.H., Wolber C., Kesimer M., Sheehan J.K., Davis C.W. (2012). Studying mucin secretion from human bronchial epithelial cell primary cultures. Methods Mol. Biol..

[278] Sedaghat M.H., Shahmardan M.M., Norouzi M., Heydari M. (2016). Effect of Cilia beat frequency on muco-ciliary clearance. J. Biomed. Phys. Eng..

[279] Smith D.J., Gaffney E.A., Blake J.R. (2007). A viscoelastic traction layer model of muco-ciliary transport. Bull. Math. Biol..

[280] Sears P.R., Yin W.N., Ostrowski L.E. (2015). Continuous mucociliary transport by primary human airway epithelial cells in vitro. Am. J. Physiol. Lung Cell. Mol. Physiol..

[281] Smith C., Radhakrishnan P., DoHyang Lee D., Hessel E.M., Williamson R., O’Callaghan C. Rhinovirus infection of human ciliated respiratory epithelial cultures. Proceedings of the 10.1 Respiratory Infections, European Respiratory Society International Congress.

[282] Feriani L., Juenet M., Fowler C.J., Bruot N., Chioccioli M., Holland S.M., Bryant C.E., Cicuta P. (2017). Assessing the Collective Dynamics of Motile Cilia in Cultures of Human Airway Cells by Multiscale DDM. Biophys. J..

[283] Mantovani G., Pifferi M., Vozzi G. (2010). Automated software for analysis of ciliary beat frequency and metachronal wave orientation in primary ciliary dyskinesia. Eur. Arch. Otorhinolaryngol..

[284] Bustamante-Marin X.M., Ostrowski L.E. (2017). Cilia and Mucociliary Clearance. Cold Spring Harb. Perspect. Biol..

[285] Button B., Picher M., Boucher R. (2007). Differential effects of cyclic and constant stress on ATP release and mucociliary transport by human airway epithelia. J. Physiol..

[286] Duncan G.A., Kim N., Colon-Cortes Y., Rodriguez J., Mazur M., Birket S.E., Rowe S.M., West N.E., Livraghi-Butrico A., Boucher R.C. (2018). An adeno-associated viral vector capable of penetrating the mucus barrier to inhaled gene therapy. Mol. Ther. Methods Clin. Dev..

[287] Liu S.L., Wang Z.G., Xie H.Y., Liu A.A., Lamb D.C., Pang D.W. (2020). Single-virus tracking: From imaging methodologies to virological applications. Chem. Rev..

[288] Liu S.L., Zhang Z.L., Tian Z.Q., Zhao H.S., Liu H., Sun E.Z., Xiao G.F., Zhang W., Wang H.Z., Pang D.W. (2012). Effectively and efficiently dissecting the infection of influenza virus by quantum-dot-based single-particle tracking. ACS Nano.

[289] Zhang L.J., Wang S., Xia L., Lv C., Tang H.W., Liang Z., Xiao G., Pang D.W. (2020). Lipid-specific labeling of enveloped viruses with quantum dots for single-virus tracking. MBio.

[290] Griffiths C.D., Bilawchuk L.M., McDonough J.E., Jamieson K.C., Elawar F., Cen Y., Duan W., Lin C., Song H., Casanova J.-L. (2020). IGF1R is an entry receptor for respiratory syncytial virus. Nature.

[291] Cohen M., Zhang X.-Q., Senaati H.P., Chen H.W., Varki N.M., Schooley R.T., Gagneux P. (2013). Influenza A penetrates host mucus by cleaving sialic acids with neuraminidase. Virol. J..

[292] Schuster B.S., Kim A.J., Kays J.C., Kanzawa M.M., Guggino W.B., Boyle M.P., Rowe S.M., Muzyczka N., Suk J.S., Hanes J. (2014). Overcoming the cystic fibrosis sputum barrier to leading adeno-associated virus gene therapy vectors. Mol. Ther..

[293] Wang S., Shan X., Patel U., Huang X., Lu J., Li J., Tao N. (2010). Label-free imaging, detection, and mass measurement of single viruses by surface plasmon resonance. Proc. Natl. Acad. Sci. USA.

[294] Neuman K.C., Nagy A. (2008). Single-molecule force spectroscopy: Optical tweezers, magnetic tweezers and atomic force microscopy. Nat. Methods.

[295] Horiguchi Y., Goda T., Matsumoto A., Takeuchi H., Yamaoka S., Miyahara Y. (2017). Direct and label-free influenza virus detection based on multisite binding to sialic acid receptors. Biosens. Bioelectron..

[296] Joyner K., Song D., Hawkins R.F., Silcott R.D., Duncan G.A. (2019). A rational approach to form disulfide linked mucin hydrogels. Soft Matter.

[297] Yan H., Hjorth M., Winkeljann B., Dobryden I., Lieleg O., Crouzier T. (2020). Glyco-modification of mucin hydrogels to investigate their immune activity. ACS Appl. Mater. Interfaces.

[298] Werlang C., Cárcarmo-Oyarce G., Ribbeck K. (2019). Engineering mucus to study and influence the microbiome. Nat. Rev. Mater..

